# Antibiotics, Analgesic Sedatives, and Antiseizure Medications Frequently Used in Critically Ill Neonates: A Narrative Review

**DOI:** 10.3390/children11070871

**Published:** 2024-07-18

**Authors:** Angeliki Kontou, Eleni Agakidou, Ilias Chatziioannidis, William Chotas, Evanthia Thomaidou, Kosmas Sarafidis

**Affiliations:** 1 Department of Neonatology and Neonatal Intensive Care, Faculty of Medicine, School of Health Sciences, Aristotle University of Thessaloniki, Ippokrateion General Hospital, 54642 Thessaloniki, Greece; eagakidou@auth.gr (E.A.); ihatzi@auth.gr (I.C.); kosaraf@auth.gr (K.S.); 2 Department of Neonatology, University of Vermont, Burlington, VT 05405, USA; 3 Department of Anesthesia and Intensive Care, Faculty of Medicine, School of Health Sciences, Aristotle University of Thessaloniki, AHEPA University General Hospital of Thessaloniki, 54621 Thessaloniki, Greece; evathom1975@yahoo.gr

**Keywords:** neonatal infections, sepsis, antibiotics, antifungal, analgesics, sedatives, seizures, medications, pharmacokinetics, neonatal pain, preterm infants

## Abstract

Antibiotic, analgesic sedative, and antiseizure medications are among the most commonly used medications in preterm/sick neonates, who are at high risk of nosocomial infections, central nervous system complications, and are exposed to numerous painful/stressful procedures. These severe and potentially life-threatening complications may have serious short- and long-term consequences and should be prevented and/or promptly treated. The reported variability in the medications used in neonates indicates the lack of adequate neonatal studies regarding their effectiveness and safety. Important obstacles contributing to inadequate studies in preterm/sick infants include difficulties in obtaining parental consent, physicians’ unwillingness to recruit preterm infants, the off-label use of many medications in neonates, and other scientific and ethical concerns. This review is an update on the use of antimicrobials (antifungals), analgesics (sedatives), and antiseizure medications in neonates, focusing on current evidence or knowledge gaps regarding their pharmacokinetics, indications, safety, dosage, and evidence-based guidelines for their optimal use in neonates. We also address the effects of early antibiotic use on the intestinal microbiome and its association with long-term immune-related diseases, obesity, and neurodevelopment (ND). Recommendations for empirical treatment and the emergence of pathogen resistance to antimicrobials and antifungals are also presented. Finally, future perspectives on the prevention, modification, or reversal of antibiotic resistance are discussed.

## 1. Introduction and Methodology

Preterm and sick neonates admitted to neonatal intensive care units (NICUs) are at high risk of developing hospital-acquired infections which are associated with severe complications, an extended need for intensive care, and increased mortality [1,2]. Factors contributing to the high incidence of infections in NICU neonates include their immature defense mechanisms, the multidrug-resistant microorganisms colonizing neonates in NICUs, and the invasive procedures they are subjected to [1]. The latter problem exposes the NICU neonates to numerous painful and stressful procedures which may have serious short- and long-term consequences [3,4]. In addition, high-risk neonates may suffer central nervous system (CNS) complications, such as peri-intraventricular hemorrhage and hypoxic–ischemic encephalopathy, which may present with seizures. All these severe and life-threatening complications should be prevented or promptly treated. To this end, evidence-based management protocols should be applied by all neonatologists. However, previous studies and reviews have revealed a wide variability in the use of medications in NICUs worldwide [3,5,6]. A major factor contributing to this variability is the lack of adequate studies regarding the effectiveness and safety of medications administered to neonates, especially the preterm ones. Reported differences primarily concern the indications and dosage of first-line medications [7].

Several studies have explored the pattern of medications used in NICUs in different countries. It was found that antimicrobial antifungals and analgesic sedatives are among the most commonly used medications in NICUs [8]. In this review, we address the antimicrobials (antifungals), analgesics (sedatives), and antiseizure medications used in neonatology, focusing on existing evidence or knowledge gaps regarding their indications, dosage, and potential side effects. We will discuss the effects of early antibiotic use on the intestinal microbiome and its association with the development of long-term immune-related diseases, obesity, and neurodevelopment (ND). The recommendations for empirical treatment and the emergence of pathogen resistance to antimicrobials and antifungals are also presented. Finally, future perspectives for the prevention, modification, or reversal of antibiotic resistance are discussed.

The literature review method included searching electronic databases (PubMed, Scopus, and the Cochrane Library) for articles on medications for the treatment of neonatal infections, pain/stress, and seizures published from January 2023 up to March 2024. Moreover, a manual search of the reference lists of the included studies was conducted to find additional relevant articles. The MeSH terms used included “acetaminophen”, “alpha-2 agonists”, “analgesics”, “antibacterial”, “antibiotics”, “antifungal”, “antimicrobial”, “antiseizure”, “benzodiazepines”, “dexmedetomidine”, “fentanyl”, “ketamine”, “levetiracetam”, “midazolam”, “morphine”, “necrotizing enterocolitis”, “neonatal infections”, “neonatal sepsis”, “neonatal pain”, “phenobarbital”, “phenytoin”, “phosphenytoin”, “placebo”, “preterm infants”, “propofol”, “randomized controlled trials”, “remifentanil”, “review”, “sedatives”, “systematic review”, and “topiramate”. The search included clinical studies (randomized clinical trials [RCTs], cohort studies, and case–control studies, either prospective or retrospective), case reports, and any kind of review studies. We included studies published as full publications in English.

## 2. Antibiotics

### 2.1. Epidemiology of Neonatal Sepsis and General Considerations for the Use of Antibiotics

Neonatal sepsis remains a significant cause of substantial morbidity and mortality in both high- and low—middle-income countries, although the precise estimates of its burden vary by setting [1,2,9]. Therefore, the prompt initiation of antibiotics in neonates with suspected or proven sepsis is very important. In this context, one out of five high-risk neonates receives at least one antimicrobial drug (92%, antibacterial; 19%, antifungal; 4%, antiviral), with “Rule-out” sepsis (32%) and “culture-negative” sepsis (16%) being the most common indications [10].

The limited available data regarding pharmacokinetics (PK) and pharmacodynamics (PD) in neonates, as well as the effectiveness and long-term adverse effects of antibiotics, have led to the off-label use of antimicrobial drugs in neonates and non-evidence based recommendations [11,12,13]. It is noteworthy that only six antimicrobials have been approved by the Food and Drug Administration (FDA) since 1998, and mainly for term infants: ceftolozane/tazobactam, clindamycin, dalbavancin, ceftaroline-fosamil, ampicillin, meropenem, and linezolid [14]. Factors that may affect the PK and PDs, and consequently the dose regimen, of these antibiotics include the degree of prematurity, postnatal age (PNA), comorbidities (perinatal asphyxia, patent ductus arteriosus, acute kidney injury), simultaneously administered medications or other interventions (i.e., extracorporeal membrane oxygenation), and genetics [15].

Depending on the time of onset, sepsis is classified into early-onset sepsis (EOS) or late-onset sepsis (LOS). EOS is defined as a culture-confirmed infection appearing in the first 3 days of life, while other authors extend this time to day 7 of life [16,17,18]. LOS occurs beyond the 3rd or 7th day of life [19,20]. Bacteria most commonly isolated from neonates with EOS are *Group B Streptococcus (GBS) and Escherichia* (*E.*) *coli*, while other Gram-positive (*Listeria monocytogene*, and *coagulase-negative Staphylococci* [*CoNS*]) and Gram-negative bacteria (*Enterobacter* spp., *Haemophilus influenzae*, *Citrobacter* spp.) are less common [1,2,9]. Fungal species, mainly *Candida* (*C.*) *albicans* and *C. parapsilosis*, occur predominantly in very low birth weight infants (VLBWIs) and represent only 1% of EOS [21,22,23].

LOS is acquired via the horizontal transmission of microbes present in the NICU environment. *CoNS* is the most commonly isolated microbe, accounting for 53–78% of LOS [24,25,26]. Other Gram-positive organisms include *Staphylococcus aureus*, *Enterococcus* spp., and *GBS* [27]. The most commonly isolated Gram-negative pathogens are *Klebsiella* spp., *Enterobacter* spp., *E. coli*, *Pseudomonas*, and *Serratia* spp., which cause 25% of LOS [28]. Fungi were reported to occur in 12% of first-time LOS episodes, with *C. albicans* being the third most frequent organism isolated (76/1313, 6%). LOS due to fungi occurs more often in preterm infants and those who received antibiotics [29,30]. Viruses are rare causes of LOS, with herpes simplex viruses being the most frequent [26].

### 2.2. Antibacterial Drugs

The antibacterials used in neonates exert their action via three main mechanisms: disruption of the bacterial cell wall (beta lactams and vancomycin); the inhibition of protein synthesis (aminoglycosides); and the inhibition of nucleic acid function (metronidazole) [31]. Challenges associated with antibacterial drug use in neonates include the antibacterial choice in relation to the isolated or potential causative bacteria and infection location, the most effective and safe dose regimen, and the duration of the treatment. The antibacterial drugs most used in neonates are discussed in the following sections.

#### 2.2.1. Penicillins—Aminopenicillins and Ampicillin

The aminopenicillin group consists of semisynthetic β-lactam antibiotics derived from benzylpenicillin after the addition of an amino group. Ampicillin is the representative member of the aminopenicillin group, which also includes amoxicillin.

Ampicillin is a broad-spectrum semisynthetic derivative of penicillin with an increased ability to penetrate through Gram-negative cell membranes. It possesses bactericidal properties due to an irreversible inhibition of transpeptidase, leading to the inhibition of bacterial cell wall synthesis and eventually cell death [32]. The antimicrobial spectrum of ampicillin includes Gram-positive microbes, such as Listeria monocytogenes, *Streptococci* spp., and *Enterococcus* spp., and susceptible Gram-negative organisms, such as strains of *Haemophilus influenzae* and *E. coli*, *Neisseria meningitidis*, *Proteus mirabilis*, and *Salmonellae*. Ampicillin is not effective against penicillinase-producing bacteria as it is hydrolyzed by beta-lactamases [31]. Ampicillin is indicated for the empirical treatment of suspected EOS including meningitis when combined with an aminoglycoside or as targeted treatment of infections, such as sepsis, pneumonia, urinary tract infections, or meningitis caused by susceptible bacteria.

PK studies have shown that the half-life of ampicillin in serum following intramuscular injection was correlated inversely with PNA [32,33], while its maximum concentration and time (Cmax and tmax) in pre-term neonates were comparable with those in older children and adults [33,34]. Moreover, early PK studies in preterm and term infants receiving multiple doses of ampicillin intramuscularly (75, 50, or 100 mg/kg/dose) showed that the concentrations in their cerebrospinal fluid (CSF) ranged significantly, being at least ten-fold higher than the maximum MICs for *GBS* and *Listeria* and equal to or greater than the MICs for *E. coli*. Of note, a synergy of ampicillin with aminoglycosides in killing *E. coli* was documented [35,36].

Based on its PK and clinical efficacy, the ampicillin dose regimen recommended by the American Academy of Pediatrics and the British National Formulary Committee [37,38] is consistently 50 mg/kg/dose bolus intravenously (IV) for sepsis, while suggested dosing intervals depend on PNA and weight, as shown in Table 1. In meningitis, higher doses—equal to or higher than 200 mg/kg/dose—are suggested since the ampicillin concentration in the CSF was found to be 11 to 65% of the neonates’ respective serum levels. However, when ampicillin is administered combined with aminoglycoside, the recommended dose is 100 mg/kg/dose IV [36,38]. Plasma concentration monitoring is not required, except for infections due to bacteria with high MICs.

The adverse effects of ampicillin include rare allergic reactions with skin rashes, fever, diarrhea, CNS excitation, or seizures reported with very large doses in adults, and prolonged bleeding time reported with repeated doses [38,39].

#### 2.2.2. Aminoglycosides—Gentamicin

The aminoglycosides gentamicin and amikacin have a wide antimicrobial spectrum, particularly against *Enterobacteriaceae*, *Staphylococcus aureus* (both methicillin-resistant staphylococcus aureus [MRSA]- and vancomycin-resistant isolates), *Pseudomonas aeruginosa*, and, to a lesser extent, *Acinetobacter baumannii*. Their ability to exert synergy with other antimicrobials, such as β-lactams, makes aminoglycosides a preferred option in combination antibiotic regimens in clinical practice [40]. According to the recent World Health Organization (WHO) AWARE classification, gentamicin belongs to the group of ACCESS antibiotics, which are characterized by good activity against a wide range of susceptible bacteria and a lower resistance potential compared to other groups of antibiotics [41]. Gentamicin combined with a beta lactam antibiotic (ampicillin or benzylpenicillin) is the recommended first choice for the empirical treatment of neonatal sepsis according to the recent WHO AWARE antibiotic book, especially for early-onset sepsis, which explains the preferred use of gentamicin in NICUs [42,43].

It is a hydrophilic drug, mainly distributed into extracellular water, with low plasma protein binding capacity, and it is mainly excreted unchanged via the renal route. In neonates, it has an increased volume distribution and prolonged elimination half-life compared to adults [44]. Despite its extensive use in neonatal sepsis, a Cochrane systematic review of RCTs has failed to prove the optimal dosing regimen in terms of efficacy, although PK data suggested that the “once a day regimen” may be superior in treating sepsis in neonates of >32 weeks’ gestation, due to higher achieved peak levels with lower toxic trough levels [45]. Further evidence supports that, due to the increased volume of distribution and reduced glomerular filtration rate in neonates, higher doses over extended time intervals (up to 48 h) are preferred [46]. Most important factors influencing PK characteristics include weight (birth weight [BW], current weight) and age (predominantly gestational age [GA] or GA combined with postnatal age) [47].

Like other antimicrobials in the field of neonatology, there is not currently a consensus dosing regimen for preterm and term neonates. Most studies, national guidelines, and neonatal drug formularies suggest an initial dose of 4–5 mg/kg, with prolonged dosing intervals of 24–48 h for preterm and term neonates [47,48,49,50,51,52,53,54]. Until recently, studies have continued to reveal that the ability of currently used dosing regimens to reach effective and safe target levels is questionable, as a significant proportion of neonates achieve either subtherapeutic or supratherapeutic concentrations [55]. Currently, a physiologically based PK-PD model for preterm and term neonates revealed that extended-interval dosing regimens (6 mg/kg every 36 and 48 h for term and preterm neonates, respectively) possess higher efficacy and lower toxicity [56]. Another PK-PD model for preterm infants that considers both the postmenstrual age (PMA) and postnatal age (PNA) suggested that a higher dose with an extended dosing interval (5 mg/kg every 36 h) in newborns with a PMA of 30–34 weeks and a PNA of 8–28 days, as well as in those with a PMA ≥35 weeks and a PNA of 0–7 days, is more likely to achieve the targeted trough concentration compared to once-daily dosing [57]. In neonatology, peak serum gentamicin concentrations of 8–12 mg/L with a trough concentration of <1 mg/L are widely applied [58]. To this end, therapeutic drug monitoring is strongly suggested for the optimization and individualization of the dose regimen in critically ill neonates.

The main adverse effects reported in neonates include nephrotoxicity, ototoxicity, and hypersensitivity (very rare), while neuromuscular blockades were reported only in adults [48].

#### 2.2.3. Carbapenems—Meropenem

The carbapenems (meropenem, panipenem, ertapenem, doripenem, and imipenem) belong to the family of b-lactam antibiotics which are resistant to hydrolyzation by most β-lactamases, including extended-spectrum β-lactamases (ESBLs) and AmpC β-lactamases (cephalosporinases). They exert their action by binding to several different bacterial penicillin-binding proteins, inhibiting their action eventually leading to cell lysis [59,60]. They possess broad bactericidal activity against Gram-negative bacteria, Gram-positive bacteria, and anaerobes; they are not active against *Enterococcus faecium*, *MRSA*, *or Stenotrophomonas maltophilia.*

Meropenem is the most commonly prescribed carbapenem for neonatal LOS in European NICUs [6]. In the most recent (2022) Infectious Diseases Society of America guidance report on the treatment of antimicrobial-resistant infections, meropenem had a significant role in the treatment of multi-resistant microbe infections [61,62]. Meropenem is mainly renally excreted, with a short elimination half-life (1 h) in normal renal function. Its ability to rapidly and effectively distribute into tissues of many organs after 1 h of IV administration makes meropenem appropriate for a variety of systematic infections, such as intra-abdominal infections and meningitis, for which the drug has been approved in adults and pediatric patients [63,64]. Meropenem exhibits a time-dependent bactericidal action [65].

In neonates and small infants <3 months old, meropenem has been FDA-approved since 2014, but only for complicated intra-abdominal infections based on non-comparative studies regarding its PK, safety, and effectiveness [66,67,68,69]. Smith et al. found that, in preterm and term infants ≤90 days old, meropenem clearance depends on their creatinine clearance and PMA. The suggested dosage regimen (20–30 mg/kg, every 8–12 h, stratified according to GA and PNA), which was eventually adopted in its FDA labeling, achieved the PK/PD target in nearly all infants [68]. Thereon, the largest European open-label, phase III superiority randomized clinical trial (RCT) in hospitalized term and preterm neonates with LOS and meningitis so far has contributed to a better understanding of meropenem’s efficacy and plasma and CSF PK [70,71].

In terms of efficacy, the Neomero-1 trial in neonates with culture-positive LOS showed that meropenem was superior to the standard of care and resulted in a shorter duration of treatment. Of note, the study was underpowered as an efficacy trial. Neomero investigators suggested that meropenem should be the preferred option for the treatment of Gram-negative LOS in severely ill neonates, mainly in NICUs where ESBL and AmpC-type beta-lactamases producing Gram-negative bacteria are common [70]. The dosing regimen used in the Neomero study was different from the FDA labeling: 20 mg/kg/dose and 40 mg/kg/dose for LOS and meningitis, respectively, over 30 min, every 12 h in preterm neonates with a GA of <32 weeks and <14 days old and every 8 h in those with a GA ≥ 32 weeks. The main findings of the Neomero population PK study were (a) in cases of LOS due to organisms with a minimum inhibitory concentration (MIC) ≤2 mg/L, the dose of 20 mg/kg bolus seemed to be sufficient, as 90% of patients achieved the PK/PD target. However, an increased dose (40 mg/kg) is warranted in cases of a MIC greater than 4 mg/L; (b) Continuous infusion resulted in increased plasma % with time over the MIC, but in lower CNS concentrations, probably because of the lower maximum concentration leading to lower peripheral penetrations; (c) meropenem’s CNS penetration significantly increased with increasing CSF protein concentration (over 40% for a CSF protein level of 6 g/L), which is indicative of inflammation of the meninges [71]. A recent physiology-based PK study in neonates showed that a favorable target attainment was achieved across all dosing groups, further supporting the dosing regimen currently recommended by the FDA [72].

Limited and low-quality data in pediatric patients and neonates regarding the mode of meropenem infusion indicate that, compared to traditional intermittent dosing regimens, continuous and extended infusion may be associated with better outcomes, i.e., clinical improvement, microbial eradication, and reduced mortality, without any difference in adverse effects [73,74,75,76].

Meropenem’s side effects include diarrhea, rash, vomiting, glossitis, neutropenia, leukopenia, and elevated creatinine, direct bilirubin, and liver enzymes [35].

#### 2.2.4. Glycopeptides—Vancomycin

Vancomycin is the most common glycopeptide antibiotic used either as empirical or targeted therapy in LOS caused by *CoNS*, *MRSA*, and *Enterococci* species in NICUs (Table 1). It is primarily renally eliminated, with a complex PK profile affecting its overall activity [77]. In addition to its PK properties, its unique neonatal physiological characteristics, such as a high extracellular water percentage, decreased renal clearance, reduced protein binding capacity, developmental immaturity, and growth, explain its higher PK variability in neonates compared to adults [78]. Vancomycin combined with an aminoglycoside or an antibiotic with an optimal penetration of the CSF is indicated as the first-line antibiotic combination used for the empirical treatment of suspected Gram-positive infections including meningitis [79].

Vancomycin exhibits time-dependent bactericidal activity against methicillin-susceptible staphylococcus aureus and *MRSA* [77]. The doses recommended by the consensus guidelines for neonates and infants <3 months old range from 10 to 20 mg/kg every 8–48 h, depending on specific covariates known to affect PK, such as PMA, body weight, and serum creatinine [80].

Despite the use and extended research performed on vancomycin for many decades, there is no consensus on its optimal utilization and monitoring in neonates. Gaps regarding the optimal dose, the mode of delivery, the duration of therapy, its index of therapeutic efficacy, and the mode of therapeutic monitoring remain. The lack of robust evidence is reflected in many different population PK models and dosing regimens recommended in neonatal formularies [11,35,81]. A recent systematic review evaluating the relationship between dosing regimens and the achievement of clinical and PK indices correlated to efficacy and safety could not reach a clear conclusion regarding the optimal therapeutic regimen due to the wide variability in study designs and endpoints [82]. Of note, the target serum concentrations used in the selected studies varied significantly, with a range of 5–30 mcg/mL [82,83,84,85].

Ramos et al. conducted an experimental study on bloodstream *CoNS* infections which showed that a higher area under the concentration/MIC targets than are currently proposed for *MRSA* infection are required for maximum effectiveness and that continuous infusions may be associated with an increased risk of the emergence of antimicrobial resistance [86]. An RCT conducted by Gwee et al. showed that continuous infusion is associated with the earlier and improved attainment of target concentrations compared with intermittent infusion, which resulted in lower total daily doses required to achieve the target levels and no safety concerns [87].

The NeoVanc study, the largest neonatal, open-label, multicenter, phase 2b, parallel-group, randomized, non-inferiority trial of vancomycin, compared the efficacy and safety of an optimized regimen (25 mg/kg IV loading dose, followed by 15 mg/kg every 12 or 8 h depending on PMA for 5 ± 1 days) to a standard regimen (no loading dose; 15 mg/kg every 24, 12, or 8 h depending on PMA, for 10 ± 2 days) in hospitalized infants ≤90 days old with LOS. Vancomycin was administered intravenously over 60 min. No clear clinical impact of the optimized regimen was demonstrated, while a potential hearing safety signal was identified in the optimized arm (30% of the infants versus 15% of the infants in the standard regimen, *p* = 0.03) [88]. A long-term follow-up study is ongoing. Data from the pharmacokinetic analysis have not been published yet.

Nephrotoxicity and abnormal hearing screening are the main adverse effects of vancomycin [89].

The indications, dosing regimens, and side effects of antibacterial agents are summarized in Table 1, while more details are presented in the Supplementary Table S1 [35,38,71,90,91,92].

**Table 1:** Selective antimicrobial drugs most often used in neonates.

Medication [References]	Mechanism of Action/Bactericidal Spectrum	Main Neonatal Indications	Neonatal Dosing Regimen	Side Effects
AMPICILLIN(a beta-lactam antibiotic classified as an aminopenicillin) [31,38,39]	Inhibition of bacterial cell wall synthesis.Bactericidal spectrum: susceptible Gram (+) (incl. *Streptococcus* spp., *Enterococcus faecalis*, *Listeria monocytogene*s) and Gram (−) bacteria (*E. coli*, *Hemophilus influenzae*, *Neisseria meningitidis*, *Proteus mirabilis*, *Salmonellae*).	Empirical and targeted treatment of suspected/proven LOS (incl. meningitis) combined with an aminoglycoside.	AAP recommendation: Septicemia: 50–75 mg/kg/dose, IV, q8–q12 for 7–28 days, depending on GA and PNA.Meningitis: 75–100 mg/kg/dose, IV, q6–q8 for ≤7–28 days depending on GA and PNA.	Allergic reactions, diarrhea, neurotoxicity including seizures, and prolonged bleeding time with repeated doses.
GENTAMICIN [35,40,42,47,49,51,55,57]	Inhibition of protein synthesis leading to cell death. Bactericidal spectrum: *Enterobacteriaceae*; *Staph. aureus* (MRSA and vancomycin-resistant isolates); *P. aeruginosa*. To a lesser extent *Acinetobacter baumannii*.	Empirical treatment of suspected EOS combined with ampicillin. Targeted treatment of infections caused by susceptible Gram (−) bacilli (e.g., *Pseudomonas*, *Klebsiella*, *E. coli*) combined with a β-lactam antibiotic.	Recommending dosages: 4–5 mg/kg/dose, dosing intervals 24–48 h depending on GA, PMA, and PNA. TDM is strongly suggested in therapy with a duration > 7 days, therapeutic hypothermia, renal impairment; target trough concentration: <2 mg/L.	Nephrotoxicity, ototoxicity, hypersensitivity (very rare), and neuromuscular blockade (reported only in adults).
MEROPENEM [35,61,71,93]	Binds to membrane proteins disrupting bacterial cell wall synthesis. Bactericidal spectrum: (i) Gram (−) pathogens *Enterobacteriaceae*, ESBL- and AmpC-producing *Enterobacteriaceae*; (ii) Gram (+) pathogens *Staph. aureus* (methicillin/oxacillin-susceptible), *Strept. pneumoniae* (incl. penicillin-resistant strains) and *Strept. viridans*; (iii) anaerobes (*Clostridium difficile*).	Severe neonatal infections (e.g., septicemia, bacterial meningitis) due to multi-drug-resistant Gram (−) organisms.	Intra-abdominal and non-CNS infections (FDA label): 20–30 mg/kg/dose, IV, q12–q8 depending on GA and PNA. CNS infection (off-label) recommended dose: 40 mg/kg/dose, IV, at q12–q8, depending on GA and PNA.	Diarrhea, rash, vomiting, glossitis, neutropenia, leukopenia, elevated creatinine, direct bilirubin, and live enzymes.
VANCOMYCIN [11,35,79,80,81,82,84,85,89,90]	Interferes with cell wall synthesis, inhibits RNA synthesis, and alters plasma membrane function.	Infections due to susceptible strains of *Staphylococcus* (incl. MRSA), *Streptococci*, *Enterococci*, *Diphtheroid*, *Listeria monocytogenes*, *Actinomyces*, and *Bacillus* spp.	Standard dose: 15 mg/kg/dose, IV, q18–q8 depending on GA and PNA. Consider loading dose 20 mg/kg/dose in cases of severe sepsis, *MRSA*, bone infection, meningitis, and endocarditis. TDM is strongly suggested more frequently in renal impairment, the use of nephrotoxic drugs, or suspected severe sepsis.	Nephrotoxicity, ototoxicity, rash and hypotension (red man syndrome), neutropenia (reported in treatment duration >3 weeks).

AAP, American Academy of Pediatrics; CNS, central nervous system; *E.*, *Escherichia*; EOS, early-onset sepsis; ESBLs, extended-spectrum β-lactamases; FDA, Food and Drug Administration; GA, gestational age; Gram (+), Gram-positive; Gram (−), Gram-negative; incl, including; IV, intravenous; LOS, late-onset sepsis; MRSA, methicillin-resistant *Staphylococcus aureus*; P., *Pseudomonas*; PNA, postnatal age; PMA, postmenstrual age; spp., species; Strept, *Streptococcus*; TDM, therapeutic drug monitoring.

### 2.3. Epidemiology of Fungal Infections in Neonates

Invasive fungal infections constitute a significant life-threatening infection in neonates, accounting for 10% of LOS in VLBWIs [19,94,95,96,97]. *C. albicans* remains the leading cause, followed by *C. parapsilosis*, *C. tropicalis*, *C. glabrata*, and *C. krusei*, whereas *C. auris* has emerged as the third most commonly encountered species causing neonatal invasive candidiasis (IC) in low—middle-income countries [98]. In a recent European 12-week modified point prevalence study (mPPS), which included 26 NICUs, 17 hospitals, and eight countries, the median percentage of neonates receiving antifungal agents per mPPS week across all Level III NICUs was 9.6% (range 7.5–11.4%). According to the GARPEC-PPS study (Global Antimicrobial Resistance, Prescribing and Efficacy in Neonates and Children-PPS), 43.5% of all antifungal prescriptions were for extremely preterm neonates (GA < 28 weeks), whereas antifungals represented 17.3% and 13.4% of all antibiotics administered in this specific group in high- and low—middle-income countries, respectively (*p* = 0.26) [99].

*C. albicans* and *Aspergillus* infections in neonates are characterized by a mortality rate of 30 to 80%, even after prompt antifungal treatment [27]. The EUROCANDY study revealed that neonates are the second most vulnerable age group to IC (IC) among pediatric patients ≤18 years old [100,101]. The incidence of neonatal IC has been reduced in the last decade in high-income countries, but the burden of fungal disease is still significant in high-risk neonates, with a mortality rate of 7.7 to 26% in pediatric patients and 20% to 50% in extremely low birth weight infants (ELBWI, [BW] < 1000 g), as well as severe neurodevelopmental (ND) sequelae [96,97,102,103,104,105,106,107]. An important factor associated with the increased incidence of fungal infections is prematurity, especially BWs < 750 g, due to related immature host defense mechanisms and immature skin and cutaneous barriers. Other predisposing factors include prolonged hospitalization, the use of central vascular catheters and parenteral nutrition, and the use of broad-spectrum antibiotics (especially glycopeptides and fourth-generation cephalosporins), skin care, and incubator humidity [32,96,98,108,109]. Most neonates with IC included in the NeoOBS invasive candidiasis sub-study (data from low–middle-income countries) had a GA > 28 weeks (81%) and BW > 1000 g (73%).

### 2.4. Antifungal Drugs Used in Neonates

Antifungal drugs used against neonatal invasive fungal infections are classified into four classes: polyenes (amphotericin Β deoxycholate [AmB-D], and its three lipid congeners), triazoles (fluconazole, voriconazole, posaconazole), echinocandins (micafungin, caspofungin, anidoulafungin), and nucleoside analogs (flucytosine) [110]. Robust evidence of a preferred first-line empirical antifungal drug for neonatal IC is lacking [111]. Thus far, there are no FDA-approved antifungal drugs for the treatment of neonatal Candidiasis with meningoencephalitis in infants less than 1 or 4 months of age [112].

#### 2.4.1. Polyenes

The polyenes used in neonates include AmB-D and its lipid formulations: liposomal AmB (LAmB), AmB lipid complex, and AmB colloidal dispersion. AmB-D or fluconazole are recommended as the first-choice treatment of systemic *Candida* infections [106]. Polyenes exert broad fungicidal activity via binding to the ergosterol of the fungal cell membrane. The polyene–ergosterol complex creates pores in the fungal cell membrane, leading to electrolyte leakage, cell lysis, and cell death [32,113]. AmB-D and LAmB have the same antifungal spectrum and are effective against most *Candida* spp., except for *C. krusei* and *C. glabrata*, as well as against *Aspergillus* spp. and *Cryptococcus* spp. [114]. Early experimental PD studies showed that AmB-D and LAmB have a fungicidal effect on *Candida* and *filamentous* fungi in a concentration-dependent manner at doses higher than their corresponding MICs [115,116]. These findings suggest that higher doses at longer dosing intervals are associated with increased effectiveness [116].

AmB-D and LAmB are indicated as a first-line therapy of invasive fungal infections with susceptible fungi, including CNS infections [106]. They are also suggested as an alternative therapy for invasive aspergillosis in neonates [106]. The currently recommended dose of AmB-D is 0.5–1.5 mg/kg/day every 24 h IV, infused over two to six hours, and LAmB’s dose is 2.5–7 mg/kg/day every 24 h IV, infused over two hours [117]. There are no data as to the duration of treatment with AmB-D. Based on expert opinions, the suggested duration for invasive fungal infections, such as sepsis and peritonitis, is 14 days after the last sterile culture and an improvement of clinical condition. For deeply localized, difficult to treat infections, such as endocarditis, osteomyelitis, etc., a treatment duration equal to or longer than 6 weeks is recommended [117,118].

Nephrotoxicity is a serious adverse effect of AmB-D therapy in adults. However, in neonates, polyene’s use is considered safer than in older children and adults [119]. Studies in VLBWIs with systemic *Candida* infections treated with either AmB or one of AmB’s lipid formulations, showed that the incidence of nephrotoxicity and hepatotoxicity were significantly higher in infants treated with AmB but comparable between those treated with either AmB-D or AmB lipid formulations [120,121,122]. On the other hand, due to the reduced renal excretion of the lipid formulation of AmB, the Infectious Diseases Society of America warns that it should be used with caution, particularly in the presence of urinary tract infections and renal fungal balls [106,123]. Consequently, the currently available data suggest that renal function should be monitored in clinical practice [119,121,124,125,126]. Other reported adverse effects, observed mainly in adults, include electrolyte (hypokalemia, hypomagnesemia, hypocalcemia) and hematological disturbances (anemia, leukopenia, thrombocytopenia), gastrointestinal side effects (elevated liver enzymes, diarrhea, vomiting), thrombophlebitis at the injection site, and general infusion-related reactions (fever, hypotension, and skin rashes). Therefore, the close monitoring of renal function, liver function, electrolytes, and full blood count is strongly suggested [106,121,123].

Several studies compared the effectiveness and safety of the different AmB formulations. Two clinical studies compared AmB-D versus LAmB. A study in 56 neonates with candidemia treated with either AmB-D, LAmB, or colloidal dispersion AmB did not show any significant difference in efficacy or safety [127]. Another study showed a higher mortality rate in neonates treated with lipid formulations of AmB compared to AmB-D. However, whether the difference in mortality was due to the different antifungal drugs or different disease severities could not be clarified [128]. Studies comparing AmB with AmB lipid derivatives administered to neonates with systemic *Candida* infections showed that all formulations had comparable effectiveness, mortality rates, and minimum side effects. Although no severe adverse effects were reported, the incidence of renal and liver toxicity was lower in the LAmB-treated group [120,121,125].

#### 2.4.2. Triazoles

Fluconazole is the representative triazole used in neonates. It exerts antifungal action via the selective inhibition of cytochrome P-450 sterol C-14 alpha demethylation, leading to the inhibition of cell membrane ergosterol synthesis and eventually to the impairment of cell membrane permeability and cell death [103,129]. The antifungal spectrum of fluconazole includes *Candida* spp. and *Cryptococcus* spp., but not *Aspergillus* spp. [130]. It is recommended as a second-line drug or a first-line drug along with AmB for the treatment of systemic *Candida* infections [106]. However, hepatotoxicity and fungi resistance to azoles may limit its use [103].

PK studies showed the larger volume of distribution and longer half-life of fluconazole in neonates compared to older children and adults. The wide distribution of fluconazole to most organs and tissues, including the CNS and kidneys, and its effective penetration into the CSF and urinary tract, make it a reasonable alternative therapeutic option for systemic *Candida* infections, provided that the neonate has not been previously on fluconazole prophylaxis [103,131,132]. In CNS infections, fluconazole is suggested as a step-down therapy after the determination of its in vitro MIC and proven clinical response to an initial therapy with AmB-D or LAmB (5 mg/kg/day) [106,133]. Growing evidence from population PK in term and preterm infants favors the use of lower doses in preterm infants compared to those labeled for term neonates, namely a loading dose of 12 to 25 mg/kg followed by 6 to 12 mg/kg/day IV or orally [111,123,134,135,136]. Higher doses are used for treating severe infections or *Candida* strains with increased MICs (4 to 8 mcg/mL) [35,38]. A physiology-based PK model investigating the CNS’s exposure to fluconazole supported the above dosing regimen, as it resulted in a more rapid attainment of the target levels in the plasma and cerebrospinal fluid of preterm infants with CNS infections [137]. Fluconazole dosing is based on the serum creatinine values and GA of the patient [111].

In addition, fluconazole is administered in high-risk VLBWIs for prophylaxis from *Candida* infections. A recent meta-analysis of nine RCTs showed that fluconazole is effective in reducing the *Candida*’s colonization rate, the incidence of IC, and its in-hospital and infection-attributed mortality, which is similar to that reported in previous systematic reviews and meta-analyses [133,138,139]. Several prophylactic dosage regimens have been studied, with different dose ranges (from 3 to 6 mg/kg), different dosing intervals (every 24–72 h), and varying durations (4 to 6 weeks) [111]. Considering that the higher dose (6 mg/kg) does not increase efficacy while potentially increasing the risk of toxicity and cost, the use of the lowest dose (3 mg/kg) seems to be preferred [140]. Both the Infectious Diseases Society of America and European Society for Clinical Microbiology and Infectious Diseases recommend fluconazole prophylaxis in high-risk neonates in NICUs with a high frequency of IC (>10%) [141]. However, the higher dose of 6 mg/kg for *Candida* spp. with a MIC >2–4 mg/L may be needed (Table 2, Supplementary Table S2) [111].

Rare adverse effects have been described for fluconazole, with hepatotoxicity and bone marrow suppression being reported the most. Specifically, the most common side effects comprise hypokalemia; hematologic, renal, and liver impairments; and gastrointestinal discomfort, while rare events of chills and fever have also been observed. Comparison studies showed that the incidence of adverse effects was lower in subjects treated with a combination of fluconazole and flucytosine than in those treated with an AmB–fluconazole combination, while no difference in effectiveness or mortality rate was observed [142,143]. A comparison between fluconazole and either AmB lipid formulations or AmB-D revealed a lower mortality rate, shorter duration of treatment, and fewer side effects in infants treated with fluconazole [128,144].

#### 2.4.3. Echinocandins

The role of echinocandins in neonatal IC is limited to salvage therapy or situations in which resistance, toxicity, or patient contraindications preclude the use of AmB-D or fluconazole [106]. However, in the context of the increasing resistance of *Candida* spp. to AmB-D and fluconazole, the role of echinocandins in the treatment of fungal infections has been upgraded. Echinocandins exert their antifungal action via the inhibition of the synthesis of the (1,3)-β-d-glucan synthase enzyme complex, leading to lysis of the fungal cell wall and cell death [112]. They possess fungicidal properties against most isolates of *Candida* (*C.*) (*C. albicans*, *C. glabrata*, *C. krusei*, *C. parapsilosis*, and *C. tropicalis*) and growth-inhibitory effects against *Aspergillus* spp. without killing them [116]. The echinocandin family includes three drugs: caspofungin, micafungin, and anidulafungin [112].

##### Micafungin

Micafungin is the most used echinocandin in neonates. It has been approved by the FDA (2019) and the European Medicines Agency (EMA) (2016) for use in infants younger than 4 months without meningoencephalitis [112]. The main indications for micafungin are invasive neonatal candidiasis with meningoencephalitis [35]. PK studies found that ELBW neonates had increased clearance compared to neonates with a BW > 1000 g [145]. However, this finding was not confirmed by another PK study [146]. A subsequent PK study by the same research team in 13 neonates showed that the doses that could attain serum levels adequate for providing CNS protection were 7 mg/kg/day for ELBWI and 10 mg/kg/day for neonates with higher BWs [147]. The FDA (2019) and the European Medicines Agency (2016) recommended a dosing regimen of 4 mg/kg/day. Target populations for this dose include stable full-term infants younger than 4 months with line-related candidemia or those with significant toxicities of other antifungal drugs [112]. Considering the difficulty of ruling out *Candida* meningoencephalitis in premature and critically ill infants at <4 months of age with candidemia and a risk of underdosing, experts appreciate that a substantially higher micafungin dose of at least 10 mg/kg once daily is likely needed for the treatment of candidemia with meningoencephalitis [112]. The rare adverse effects of micafungin in term and preterm infants include elevated serum levels of liver enzymes, temperature elevation, hypokalemia, and hyperbilirubinemia [32,98,145,146,147]. Comparison of the safety and effectiveness between IV micafungin and IV AmB was carried out in a phase 3, randomized, double-blind, multicenter study. Thirty (30) infants with a PNA of 3–120 days (20 in the micafungin group and 10 in the AmB-D group) with proven IC were recruited. However, the study was terminated early because of slow recruitment. The available results showed that the infants in the two study groups demonstrated a comparable fungi-free survival and treatment-associated adverse events, including mainly anemia and thrombocytopenia. Both drugs were well tolerated [147].

##### Caspofungin

Caspofungin has been approved by the FDA only for infants >3 months of age, as data concerning its use in neonates and small infants aged less than 3 months are limited [148,149,150,151]. Like micafungin, in vitro studies showed that caspofungin exerts fungicidal action against *Candida* spp. and fungistatic action against *Aspergillus* spp. [148]. An RCT compared the efficacy, safety, and tolerability of caspofungin versus AmB-D in neonates with IC. Thirty-two neonates with IC received either caspofungin (*n* = 15) or AmB-D (*n* = 17). It was found that caspofungin is more effective and safer and could be used as an alternative to AmB-D for the treatment of IC in neonates [152]. A more recent multicenter, Phase 2 RCT compared the efficacy of caspofungin versus AmB-D in infants aged less than 3 months with culture-confirmed IC. Of the 49 enrolled neonates, 33 received caspofungin and 16 AMB-D. However, the study was terminated due to slow recruitment. The available results showed that the infants in the two study groups achieved comparable fungi-free survival. The prevalence of at least one treatment-associated adverse event was 84.8% in the caspofungin group versus 100% in the AmB-D group. The most frequent adverse effects were edema at the infusion site and cholestatic jaundice in the caspofungin group and anemia, increased blood lactate dehydrogenase level, and metabolic alkalosis in the AmB-D group. A higher proportion of the AmB-D group (48% versus 18%) developed severe adverse effects including cholestasis, endocarditis, accidental overdose, or superior vena cava syndrome, as well as cardiac arrest and procedural pneumothorax in one AmB infant [151].

##### Anidulafungin

Anidulafungin is the most recently developed echinocandin. It is currently approved for infants >1 month of age due to limited data in neonates. The mechanisms underlying its antifungal effects and antifungal spectrum are the same as the other echinocandins [153,154]. However, anidulafungin was found to be the most potent echinocandin against *Aspergillus fumigatus* spp. [155]. A dosing regimen of 1.5 mg/kg/day, derived from experimental PK studies and translated to humans, was recommended for neonates with proven or suspected *Candida* meningoencephalitis. A PK study showed that a loading dose of 3.0 mg/kg and daily maintenance dose of 1.5 mg/kg/day in neonates and infants less than 2 years old corresponded to an anidulafungin exposure level similar to that in adult patients receiving 100 mg/day [154]. Similar results were reported by a more recent PK study including six infants (0.1–21.8 months age) with IC treated with anidulafungin for 5–3 days (3 mg/kg on day 1, 1.5 mg/kg daily thereafter). However, an experimental PK study reported that the proposed dosing regimen may not be sufficient to treat *candida* meningoencephalitis and that higher dosages are required for maximum antifungal effect [156,157]. The main side effects of anidulafungin included fever, hypotension, headache, exanthem, dizziness, nausea, diarrhea, hypokalemia, elevations of liver enzymes, neutropenia, and leukopenia. Most side effects are dosage-dependent [153]. In a more recent study, 19 infants, 1 month to less than 2 years of age, with microbiologically confirmed IC (*n* = 16) or at high risk of IC (*n* = 3) were treated with anidulafungin for 5–35 days at the recommended doses. Overall, the success rate was 68.8%, while only mild/moderate adverse effects were reported and no treatment-related deaths [158].

#### 2.4.4. Nucleoside Analogs—Flucytosine

Flucytosine (5-fluorocytosine or 5FC) is a synthetic fluorinated nucleoside analog of cytosine possessing antifungal properties. It has been licensed by the FDA as an antifungal drug since 1974 [159]. The 5FC is a pro-medication that is deaminated by the fungal enzyme cytosine deaminase to 5-fluorouracil (5FU), which is the active form of the drug exerting antifungal activity. The 5FU undergoes successive metabolic alterations eventually leading to the inhibition of protein and DNA synthesis [160]. 5FC is safe in humans at pharmacological doses because human cells do not possess the cytosine deaminase enzyme. However, side effects may occur at high doses or in the setting of renal dysfunction where its plasma levels can increase to over 100 mg/mL. Such increases have also been attributed to conversion of 5FC to 5FU by the gut microbiome [159]. Flucytosine has a good penetration into the urinary tract, CNS, eye, cardiac vegetations, and fungal biofilms. Its antifungal spectrum includes *Cryptococcus* spp., *Candida* spp., and *dematiaceous fungi*. The main indications for flucytosine include Cryptococcal meningitis and systemic Candida infections caused by *Candida* spp. resistant to azoles. Flucytosine should always be used in combination with other antifungal agents, such as AmB, to prevent a rapid development of resistance [161].

Data in neonates are very rare and limited to a few case reports [35]. Thus far, two early studies have been published. Smith et al. reported a case series with eight extremely preterm and two term neonates (PNA range of 16 to 58 days) with systemic candidiasis. Treatment with flucytosine alone was implemented in all neonates at a dose of 100 to 200 mg/kg/day. Four infants did not respond to flucytosine monotherapy and received additional AmB-D. The combination of flucytosine and AmB-D successfully treated the infection. Four neonates died, but their death was not attributed to candidiasis or the treatment [162]. McDougall et al. reported two cases of systemic candidiasis in ELBWIs who did not respond to IV miconazole, but they responded to a combined treatment with AmB and 5-fluorocytosine [163]. The reported adverse effects include bone marrow suppression with anemia, leukopenia, thrombocytopenia, and hepatotoxicity, which usually occur with plasma concentrations higher than 100 mg/L; hypokalemia; acidosis; diarrhea; nausea; vomiting; and exanthem [164,165].

The indications, dosing regimens, and side effects of antifungal agents are summarized in Table 2, while more details are presented in Supplementary Table S2. An executive summary is shown in Table 3.

**Table 2:** Selected antifungal medications most often used in neonates.

Medication [References]	Mechanisms of Action/Fungicide Spectrum	Main Neonatal Indications	Neonatal Dosing Regimen	Side Effects
Amphotericin B Deoxycholate (AmB-D) (Polyene) [32,106,110,113,116,117,120,121,124,128]	Loss of cell membrane integrity by binding to ergosterol. Potent and broad fungicidal activity.	Invasive fungal infections of susceptible *Candida* spp., *Aspergillus* spp., and *Cryptococcus* spp. First-line therapy for neonatal IC including CNS infections.	First-line treatment: 1 mg/kg, IV, q24. Step-down treatment of CNS infections: 5 mg/kg, IV, q24	Nephrotoxicity (especially in co-adm. with other nephrotoxic drugs), electrolyte disturbances, anemia, leukopenia, thrombocytopenia, elevated liver enzymes, diarrhea, vomiting, thrombophlebitis at the injection site, infusion-related reactions (fever, hypotension, skin rashes). Monitoring: renal and liver function, electrolytes, and full blood count.
Liposomal Amphotericin B (LAmB-D) (Polyene) [32,110,113,128]	Same as Am B-D	Same as AmB-D. Alternative therapy for neonatal IC (caution in renal infection or dysfunction). Drug of choice for invasive aspergillosis.	3–5 mg/kg, IV, q24. Step-down treatment of CNS infections: 5 mg/kg, IV, q24.	Similar adverse events with AmB-D, but reduced incidence. Monitoring: renal and liver function, electrolytes, and full blood counts.
Fluconazole (Triazole) [103,106,129,130,131,132,134,136,141]	Inhibition of fungal cytochrome P450 activity and ergosterol synthesis, leading to cell membrane disruption.	Treatment of invasive infections of susceptible *Candida* spp. An alternative therapy of IC in neonates who have not been on fluconazole prophylaxis. A step-down treatment of *C. meningitis*. Prophylaxis of *C.* infections.	LD: 25 mg/kg, MD: 12 mg/kg once a day. Prophylaxis: 3–6 mg/kg every 72 h for 4–6 weeks.	Most common adverse effects: Gastrointestinal irritation and elevation in liver tests. Rare: Rash, leukopenia, neutropenia, agranulocytosis, and thrombocytopenia. Weekly monitoring of SGOT, SGPT, and ALP.
Micafungin (Echinocandin) [98,112,145,146,147,165]	Inhibition of beta (1–3)-glucan synthase activity preventing synthesis of the fungal cell wall. Fungicidal spectrum: *Candida* spp. including resistance to fluconazole spp.	Salvage therapy of IC or where resistance or toxicity preclude the use of AmB-D or fluconazole. There are concerns regarding the penetration of echinocandins into the CSF.	4 to 10 mg/kg/day, IV. Higher dose (≥10 mg/kg, q24) is likely needed for candidemia with meningoencephalitis.	Most common adverse events: infusion reactions and transient elevation of hepatic enzymes. Electrolyte disturbances, elevated creatinine, acute intravascular hemolysis, hemolytic anemia and hemoglobinuria, monocytosis, thrombocytopenia, fever, rash, diarrhea, and vomiting.

AmB-D, amphotericin Β deoxycholate; *C.*, *Candida*; CNS, central nervous system; CSF, cerebrospinal fluid; h, hours; IC, invasive candidiasis; IV, intravenous; LAmB, liposomal AmB; LD, loading dose; MD, maintenance dose; spp., species.

### 2.5. Empirical Treatment of Infections

The non-specific manifestations of neonatal sepsis that overlap with other neonatal diseases and the poor positive predictive value of laboratory tests contribute to the delayed or under-diagnosis of sepsis. However, the severe complications and high mortality of sepsis impose the early implementation of empirical treatment in infants at high risk of sepsis or suspected to have culture-negative sepsis. The general principle is that empirical therapy should be guided by the epidemiology of EOS and LOS, as well as local antimicrobial resistance patterns [1]. Suspected or high-risk EOS is the primary indication for empirical therapy in neonates. The American Academy of Pediatrics and WHO recommends ampicillin plus gentamicin as the first-line antibiotic combination for the empirical treatment of EOS. In cases with a strong clinical suspicion of severe sepsis or Gram-negative meningitis, a third- or fourth-generation cephalosporin can be administered either as a second-line agent or added to the empirical regimen [22,79,166,167]. The recommended antimicrobials are effective against the most isolated microbes from EOS cases, i.e., *GBS* (and other Gram-positive microbes) and *E. coli*. In fact, the Antibiotic Resistance and Prescribing in European Children (ARPEC) study conducted in 226 hospitals (41 countries), including NICUs, showed that the combination of ampicillin, amoxicillin, or benzylpenicillin with an aminoglycoside was the most frequently used regimen for neonatal sepsis [31,167,168]. However, there are concerns regarding the increasing resistance of *E. coli* to the currently recommended antibiotics for empirical treatment [169,170]. Therefore, modification of the empirical treatment is suggested according to the MIC of the bacteria isolated from blood cultures. In NICUs with an infection or colonization by *MRSA* at a rate higher than 10%, vancomycin is the medication of choice for empirical treatment [171].

Several studies have assessed the effectiveness of the ampicillin plus gentamicin regimen. A crossover study comparing ampicillin versus penicillin combined with gentamicin in the empirical therapy of ELBWIs at risk of EOS showed the similar effects of the different antibiotics on 72 h and/or 7-day all-cause mortality [172,173]. In agreement, an early Cochrane systematic review which included 19 trials enrolling 1496 patients of any age and sex with community-acquired acute bacterial meningitis compared the effectiveness and safety of third-generation cephalosporins with conventional treatment including penicillin or ampicillin alone or combined with chloramphenicol, with or without gentamicin. There were no significant differences between the groups concerning the risk of death, deafness, or treatment failure. However, the cephalosporin group had a significantly lower risk of positive CSF cultures after 10 to 48 h of treatment and a higher prevalence of diarrhea episodes compared to the conventional treatment group [174]. A more recent systematic review, including five RCTs with 865 infants, assessed the effectiveness and side effects of five different antibiotic regimens administered to neonates younger than 72 h PNA with EOS. No difference in mortality or adverse effects between the compared antibiotics or combinations was found, probably due to the high risk of systematic errors and a lack of adequate power [16]. In this context, the Global Antibiotic Research and Development Partnership developed alternative empirical antibiotic regimens that fulfill certain criteria. The working group identified five antibiotics as candidates for the empirical treatment of neonatal infections caused by multidrug-resistant bacteria: amikacin, tobramycin, fosfomycin, flomoxef, and cefepime. The authors commended that these five agents have the potential to be used in novel empirical regimens for neonatal sepsis in middle–low-income countries, provided that further PK and PD studies further define their characteristics [91]. At the same time, a systematic review including a total of 49 articles assessed whether the WHO-recommended regimen for the empirical treatment of LOS caused by *Enterobacteriaceae* remains applicable. It was found that the sensitivity of *Klebsiella* spp., *E. coli*, and *Enterobacter* spp. to the antibiotics recommended by the WHO was low. These data underline the need for a revision of WHO guidelines [28]. In fact, the National Institute for Health and Care Excellence (NICE) recommends the use of benzylpenicillin instead of ampicillin, while a combination of narrow-spectrum antibiotics (such as intravenous flucloxacillin plus gentamicin) is recommended for LOS as well [175]. Third-generation cephalosporins, such as cefotaxime, or fourth-generation cephalosporins should be reserved for suspected Gram-negative meningitis. Moreover, a recent systematic review evaluating the effectiveness and safety of empirical antibiotic regimens for necrotizing enterocolitis (NEC) included five studies (two RCTs and three observational) with 3161 participants. It was found that no antimicrobial regimen was superior to ampicillin and gentamicin in decreasing mortality and preventing clinical deterioration in NEC, while metronidazole could be added in patients with surgical NEC [176].

### 2.6. Long-Term Adverse Effects of Early Antibiotic Use in Neonates

Apart from the acute adverse effects of the individual antibiotics presented in the previous sections, there are major concerns regarding the long-term effects of early antibiotic treatments on the emergence of microbial resistance, changes in the intestinal microbiome, and increased infection occurrence [98]. Moreover, the potential long-term effects on ND, obesity, and immune-related diseases later in life are discussed.

#### 2.6.1. Early Factors Affecting the Neonatal Intestinal Microbiome and Their Consequences

##### Neonatal Intestinal Microbiome’s Characteristics and Functions

The intestinal microbiome is established in early life, i.e., during intrauterine life, delivery, and early postnatal days. During vaginal delivery, neonates are colonized mainly by *Bacteroides* and *E. coli* derived from the maternal microbiome [25,177]. The normal intestinal microbiome of the neonate has many beneficial effects on the developing gastrointestinal system, including the maturation of epithelial function and nutrient digestion, as well as on the development of the gut’s innate immune defense mechanisms [177,178,179]. An abnormal microbiome, also known as dysbiosis, is defined as an imbalance between beneficial and pathogenic gut bacteria. It is characterized by low diversity and a predominance of pathogenic *Enterobacteriaceae* and has been associated with sepsis and NEC, along with other adverse outcomes [25,180]. The mechanism underlying this association is the disruption of intestinal mucosal permeability to microbes by pathogenic bacteria, which is followed by bacterial translocation and subsequent excessive inflammation and abnormal immune responses [181]. The relationship between dysbiosis and sepsis/NEC is supported by several studies showing that changes in the intestinal microbiome precede sepsis and NEC in preterm infants [182,183,184]. Important factors that may influence neonatal intestinal microflora composition include the mode of delivery (vaginal delivery or cesarean section (CS)), the mode of feeding (maternal milk or formula), exposure to antibiotics, and length of NICU stay [179,185,186].

##### Effect of Birth and Feeding Mode on Microbiome and Adverse Outcomes

Birth via CS promotes the establishment of a microbiome with decreased diversity, in which proteobacteria predominate. In addition, CS has been associated with an increased rate of long-term morbidities including chronic inflammatory diseases (asthma, allergy, inflammatory bowel disease [IBD]), infectious diseases (bronchiolitis, otitis, and gastroenteritis), impaired metabolism and immune function, cardiovascular diseases, and increased hospitalizations for gastrointestinal diseases [187,188,189,190,191,192]. Moreover, a large study of 132,054 deliveries showed that elective CS is associated with adverse long-term ND outcomes including abnormal movement, sleeping, eating, myopathy, cerebral palsy, and others [188]. Bäckhed et al., using a metagenomic analysis of fecal samples from a large cohort of Swedish infants and their mothers, provided evidence of an interplay between gut microbiota and delivery mode, suggesting that chronic inflammation and abnormal immunological responses may underlie the association between delivery mode and the intestinal microbiome [193]. Additional studies further support the role of delivery mode as a significant contributing factor to the microbiome’s composition [185,194] and the rate of infections during childhood and adolescence, as well as to ND outcomes [186,189]. Of note, the inclusion of siblings minimized the potentially confounding effects of family, environmental, and genetic factors, further supporting the role of delivery mode in long-term morbidity [186].

The mode of feeding is another most important factor contributing to the establishment of a normal intestinal microbiome. In breast-fed neonates, the microflora that colonized the neonate during vaginal delivery is substituted by a normal microbiome consisting of *Bifidobacterium* spp. and *Lactobacilli* spp. [195]. Unlike breastmilk feeding, formula feeding promotes a microbiome consisting of potentially pathogenic bacteria and is associated with sepsis, NEC, and other short- and long-term adverse effects [184,194,195].

#### 2.6.2. The Role of the Early-Life Use of Antibiotics in the Fetal/Neonatal Microbiome and Associated Outcomes

##### Early Antibiotic Exposure and Sepsis/NEC

Many studies outline the role of perinatal and early-life antibiotic exposure in changes in the fetal and neonatal microbiome to include potentially pathogenic bacteria [194,196]. However, the study design does not always allow for discrimination between the effect of antibiotics from that of sepsis/NEC [197]. To this end, studies by Ting et al. investigated the association of early antibiotic administration with mortality and morbidity in a large study of 11,669 VLBW infants without culture-proven sepsis or NEC from the Canadian Neonatal network. It was found that a 10% increase in antibiotic use rate was associated with increased odds of mortality and major morbidities (chronic lung disease, persistent periventricular echogenicity or echolucency, or stage 3 retinopathy of prematurity) [25,198]. Moreover, prolonged antibiotic use in VLBWIs has been associated with abnormal findings in brain ultrasounds, an increased incidence of bronchopulmonary dysplasia, and retinopathy of prematurity, even in the absence of blood culture-proven sepsis or NEC [198,199].

##### Association of Early Antibiotic Treatment with Immune-Mediated Diseases and Obesity

Changes in the intestinal microbiome secondary to pre- and post-natal antibiotics, or other conditions, were associated with the emergence of long-term immune-mediated diseases, such as asthma and IBD (ulcerative colitis and Crohn’s disease) [200,201,202,203,204,205,206].

Several studies associated early antibiotic use with asthma and allergy in childhood and adolescence [201]. These associations were not affected by the time of antibiotic exposure, suggesting an important role of the early-life intestinal microbiome in the development of childhood asthma [202,203,207]. Two early systematic reviews showed that antibiotics slightly increased the risk of childhood asthma. The authors stated that *“truly indicated antibiotics should not be withheld from infants or young children for fears they might develop asthma*.” [208,209]. Of note, in sibling analyses, the association between antibiotics and asthma decreased or disappeared, indicating a potentially confounding effect of shared familial factors and respiratory infections [210].

Of interest is the reported association of early prolonged antibiotic administration with chronic inflammatory intestinal diseases through the antibiotic’s interaction with intestinal mucosa immune properties. Dysbiosis seems to be the link between antibiotic use and IBD. In fact, two experimental studies provided evidence of the interaction of antimicrobials with intestinal microbiome-induced autoimmune diseases [211,212,213,214].

Clinical studies evaluating the relationship between early antibiotic exposure and IBD showed that exposure to antibiotics throughout childhood was associated with the development of IBD in a dose-dependent manner. However, this association decreased with increasing age at exposure [200,204,205,206,213]. Moreover, a population-based case–control study demonstrated that antibiotic use was associated with histological findings of celiac disease. The authors concluded that the positive association between antibiotic use and lesions that may represent early celiac disease suggests that intestinal dysbiosis may play a role in the pathogenesis of celiac disease [206].

Other studies associated childhood overweight and obesity with long-term or repeated antibiotic exposure both prenatally and in early childhood, as well as with the maternal microbiome and birth via CS [215,216]. A recent systematic review by Baron et al. identified five relevant studies. All of them reported positive trends between prenatal antibiotic exposure and overweight/obesity in childhood, while the results concerning postnatal antibiotic use were controversial [217]. Moreover, a very recent longitudinal cohort study that analyzed data on 8880 mother–child pairs did not demonstrate any significant association between CS or the induction of labor with overweight, obesity, or body fat percentage and implied that previously reported results could be attributed to non-identified confounding factors [218]. Although the pathophysiology of this association is not fully understood, experimental and human studies suggest a potential role of the microbiome in metabolism changes, especially during critical periods for metabolic programming, such as fetal life and the first year of life [196,216,219]. The potential association between early-life antibiotic exposure and obesity in children is discussed in an editorial by Aza and Owora. The authors stated that “*it remains unclear whether antibiotics causally influence obesity development in humans and whether particular antibiotic types or time windows of exposure are especially detrimental. …… while RCTs including neonates receiving antibiotics without underlying disease is unethical, both antibiotic stewardship programs and childhood obesity prevention programs are clearly needed.*” [216,220].

##### Association of Early Antibiotics with Neurodevelopment

A major concern regarding the early use of antibiotics during sensitive developmental periods of extrauterine life is the potential impairment of the immature CNS and, eventually, the emergence of ND impairments. The potential mechanisms underlying this relationship have not been fully clarified. It is well known that the prenatal period is probably the most critical exposure window regulating brain development. In this respect, the concurrent development of the gut microbiota and brain may indicate the existence of gut–brain cross-talk that could affect the prescriptive path of brain development, predisposing a person to ND and behavioral defects [221,222,223]. Moreover, the early colonization of the infant gut is influenced by antibiotic use, the maternal microbiota, and other early-life exposures that may affect later microbiota diversity and ultimately ND [221,224]. Several studies associated neonatal sepsis and NEC treated with antibiotics with adverse ND outcomes. However, data regarding the clear association of early antibiotic exposure with ND in the absence of sepsis/NEC are sparse and contradictory [225,226,227].

To distinguish the association of early antibiotic treatment with long-term ND outcomes from the confounding effect of sepsis/NEC, Ting et al. designed a large cohort study involving 14,207 VLBWIs without culture-proven sepsis who received antibiotics during their first week of life. It was demonstrated that prolonged antibiotic exposure was associated with higher odds of the composite outcome, defined as mortality and any major morbidity (including severe neurologic injury). Severe neurologic impairment developed in 2%, 5%, and 10%, of the infants treated for 0, 1–3, and 4–7 days, respectively [199]. Baumfeld et al. conducted a retrospective cohort study involving children and adolescents up to 18 years of age with neurological morbidity. Their recorded neurologic adverse effects included abnormal movements, sleeping, eating, and developmental disorders, as well as myopathy and cerebral palsy. It was documented that CS was an independent risk factor for pediatric neurological hospitalization. One of the potentially underlying mechanisms may involve the abnormal microflora in the gastrointestinal and respiratory tracts secondary to antibiotic treatment and/or CS, or other neonatal factors that induce chronic inflammation and abnormal immunological responses. Thus, this study provided only indirect evidence of the potential association of antibiotic treatment with ND outcomes [188]. Bedetti et al., in a recent retrospective case–control study including VLBWIs and/or neonates of a GA ≤ 30 weeks with sepsis (*n* = 76) matched with infants without sepsis (*n* = 76), demonstrated that severe functional disability at the age of 24 months was associated with intraventricular hemorrhage (Odds Ratio 4.7, Confidence Intervals 1.7–13.1, *p* = 0.002) and all sepsis and culture-proven sepsis events. Moreover, culture-negative sepsis was not associated with an increased risk of severe functional disability compared to infants without sepsis. However, the potential effect of antibiotics on ND cannot be evaluated, since the empirical antibiotic treatment used and its duration was not clarified [228]. A study in 6565 ELBWIs showed that EOS was associated with an increased risk of death/ND impairment, while antibiotic administration to matched non-septic infants was not associated with an increased risk of death/ND impairment. [197].

Data on antifungal drugs and ND are almost entirely missing [229]. To the best of our knowledge, only two studies have examined the effect of fluconazole administration on ND. An RCT by Kaufman et al. in children aged 8–10 years who had received fluconazole for *Candida* prophylaxis during a NICU stay versus placebo (*n* = 17; age 9.3 to 0.8 years) did not identify any fluconazole-associated long-term ND impairment or effect on quality of life in these children [230]. Similar results were reported by Benjamin et al. in ELBWIs (BW < 750 g) treated with fluconazole versus placebo [133]. The results of these two studies were analyzed in a Cochrane review that reached the conclusion that “*The longer term neurodevelopmental consequences for infants exposed to this intervention remain to be determined*” [139]. Two additional Cochrane reviews could not reach a conclusion as to the effect of antibiotics on ND [222,223].

Collectively, these data demonstrate the lack of supporting evidence and outline the need for well-designed prospective studies on long-term associations between early antibiotic use and ND in the absence of important confounding factors, such as sepsis/NEC and other comorbidities. Published results were analyzed in three systematic reviews which stated that the available data are very limited and no conclusion could be drawn [139]. Two additional Cochrane reviews could not reach a conclusion as to the effect of early antibiotic use on ND [222,223].

### 2.7. Pathogen Resistance to Antibiotics

A crucial issue neonatologists should be aware of concerns the increasing rate of pathogen resistance to antibiotics [231]. Surveillance of culture-positive bacterial EOS showed that 78% of the *E. coli* isolates were resistant to ampicillin and 10% were resistant to gentamicin [170,232]. A study in middle-low-income countries showed that 97.2% of Gram-negative isolates were resistant to ampicillin and 70.3% of them were resistant to gentamicin. These data question the use of ampicillin–gentamicin as an empirical treatment for neonatal sepsis in low–middle-income countries [233]. In this line, a systematic review including a total of 49 articles found that the sensitivity of *Klebsiella* spp., *E. coli*, and *Enterobacter* spp. to antibiotics recommended by the WHO was low [28]. The increasing prevalence of *E. coli* and other Gram-negative pathogens that are resistant to ampicillin and gentamicin underlines the need for recommendation revisions [37,55,234]. Third-generation cephalosporins, such as cefotaxime, or fourth-generation cephalosporins should be reserved for suspected Gram-negative meningitis. Nevertheless, their overuse should be avoided, as their administration has been associated with the emergence of multidrug-resistant organisms and candidiasis. In this context, the Global Antibiotic Research and Development Partnership developed alternative empirical antibiotic regimens that fulfill certain criteria. Based on the suggested criteria, five antibiotics were identified as candidates for the empirical treatment of neonatal infections caused by multidrug-resistant bacteria [91].

**Table 3:** Executive summary of selected antibacterial and antifungal drug data. Dose regimens are shown in Table 1 and Table 2.

The bacteria most isolated from EOS cases are *GBS and E. coli,* while other Gram-positive and Gram-negative bacteria are less often observed [232]. The most frequently isolated microbes from LOS include *CoNS* (53–78% of LOS) and other Gram-positive (*Staphylococcus aureus*, *Enterococcus* spp.) and Gram-negative pathogens (*Klebsiella* spp., *Enterobacter* spp., *E. coli*, *Pseudomonas*, and *Serratia* spp.) [24,25,26] Fungal species, mainly *C. albicans* and *C. parapsilosis*, occur predominantly in VLBWIs and represent 1% of EOS and 10% of LOS in this population [21,22,23]. The antibiotics most often used in neonates include ampicillin, gentamicin, meropenem, and vancomycin. Ampicillin and gentamicin are indicated as first-line antibiotics for the empirical treatment of suspected EOS, including meningitis, or as a targeted treatment of infections caused by susceptible bacteria. There is no current consensus on gentamicin dosing in preterm and term neonates. The most often suggested regimen includes an initial dose of 4–5 mg/kg, followed by doses every 24–48 h for preterm and term neonates [47,48,49,50,51,52,53,54]. Meropenem is the most frequently prescribed carbapenem for neonatal LOS in European NICUs and it is effective against multidrug-resistant Gram-negative bacteria. Meropenem is well-tolerated with relatively mild side effects. Vancomycin is effective against neonatal infections caused by methicillin-susceptible and methicillin-resistant *Staphylococcus aureus*, but there are still gaps in our knowledge, particularly concerning the optimum dosage regimen and its nephrotoxicity and long-term effects. Factors associated with the increased incidence of fungal infections are prematurity, especially a BW < 750 g, due to immature host defenses; prolonged hospitalization; central vascular catheter placement; parenteral nutrition; and the use of broad-spectrum antibiotics. Antifungal drugs used against neonatal invasive fungal infections are classified into four classes: polyenes, triazoles, echinocandins, and nucleoside analogs [110]. Thus far, there are no FDA-approved antifungal drugs for the treatment of neonatal Candidiasis with meningoencephalitis in infants less than 1 or 4 months of age [112]. The choice of the most appropriate antifungal drug for an empirical or targeted treatment is based on local epidemiology data, the antifungal medication’s PK and PDs, and safety data. Of the polyenes, AmB-D and LAmB are indicated as a first-line therapy for invasive fungal infections, including CNS infections, and as an alternative therapy for invasive aspergillosis in neonates [106]. Of the triazoles, fluconazole is the one most often used. It is recommended as a second-line drug, or a first-line drug along with AmB, for the treatment of systemic *Candida*l infections. Echinocandins are fungicides against most *Candida* isolates and fungistatic against *Aspergillus* spp. They are indicated as salvage therapy, or in situations in which resistance or toxicity or patient contraindications preclude the use of AmB-D or fluconazole and as treatments for IC with meningoencephalitis [35,106,112]. Flucytosine is indicated for systemic and CNS infections caused by *cryptococcus* spp. and *Candida* spp. resistant to azoles. It is administered usually combined with AmB or other antifungal drugs to prevent the rapid development of resistance. Currently available data indicate a transient association of fetal and early postnatal antibiotic administration with immune-related diseases (asthma, allergy, and IBD) and overweight/obesity later in life. A major mechanism underlying these long-term effects is the abnormal composition and diversity of the gut microbiome, which induces chronic inflammation and abnormal immune responses, eventually leading to immune-related diseases. Theoretically, early antibiotic use may adversely affect long-term ND via dysbiosis and gut–brain crosstalk. Existing data are sparse and controversial. Well-designed prospective studies on long-term associations between early antibiotic use and ND in the absence of important confounding factors, such as sepsis and NEC, are urgently needed.

AmB-D, amphotericin Β deoxycholate; BW, birth weight; *C.*, *Candida*; CNS, central nervous system; *E.*, *Escherichia*; EOS, early-onset sepsis; GBS, *Group B Streptococcus*; IBD, inflammatory bowel disease; IC, invasive candidiasis; LOS, late-onset sepsis; ND, neurodevelopmental; NICU, neonatal intensive care unit; PD, pharmacodynamics; PK, pharmacokinetics; spp., species; VLBWIs, very low birth weight infants.

Another challenge physicians face concerns infections due to Gram-negative bacteria producing ESBLs, such as *Klebsiella pneumoniae*, *E. coli*, *Enterobacter* spp., *Salmonella* spp., *Proteus* spp., *Serratia marcescens*, and *Pseudomonas aeruginosa*, which are resistant to antibiotics containing the beta-lactam ring. Infections due to ESBL-producing Gram-negative bacteria require treatment with carbapenems, such as meropenem [61]. Treatment with piperacillin–tazobactam or ampicillin–sulbactam is being used increasingly in NICUs. However, the penetration of tazobactam into the CNS is questionable and should not be used for the treatment of meningitis. On the other hand, the β-lactamase inhibitor sulbactam, when combined with ampicillin, seems to achieve high concentrations in cerebrospinal fluid [1,235]. The use of vancomycin in the empirical therapy of LOS is based on the predominance of *CoNS* and concerns regarding *MRSA* infections. Nevertheless, there are several arguments against its use in empirical regimens due to the risk of ototoxicity and nephrotoxicity [90,171].

In this era of antimicrobial resistance, neonatal LOS due to multidrug-resistant bacteria has become a significant issue in many NICUs worldwide [61]. In the Neonatal Antimicrobial Resistance Research Network, the resistance rates of Gram-negative isolates to cephalosporins ranged from 26% to 84% and to carbapenem from 0% to 81%, while glycopeptide resistance rates among Gram-positive isolates ranged from 0% to 45% [236]. Higher mortality and morbidity are attributed to multidrug-resistant organisms compared to non-multidrug-resistant organisms causing neonatal sepsis, with case fatality rates for carbapenem-resistant organisms reaching 36% [70,169,237,238]. The Infectious Diseases Society of America has updated guidelines for the treatment of infections caused by ESBL- and AmpC beta-lactamase-producing pathogens [62,238]. However, neonates are not included in these guidance reports. A recent systematic review showed that colistin in combination with other antimicrobials, such as meropenem, amikacin, ciprofloxacin, or tigecycline, was used for infections due to carbapenem-resistant *Enterobacterales* and extensively drug-resistant *Pseudomonas aeruginosa*.

In addition to increasing antibacterial drug resistance, the continuous emergence of fungal resistance against commonly used antifungal drugs is an additional source of serious concern worldwide [239,240]. Fungal resistance to antifungal drugs may be intrinsic or acquired [158,241,242]. Intrinsic resistance is attributed to several factors, including biofilm formation and cell wall impermeability, while genetic factors have also been implicated in the development of intrinsic or mixed (i.e., intrinsic and acquired) antifungal drug resistance [243,244]. The most important predisposing factor for acquired resistance is exposure to fungistatic antifungal agents, especially at sub-therapeutic concentrations [245]. *Candida* spp. may develop resistance to azoles and echinocandins that are often used for prophylaxis or the empirical or targeted therapy of *Candida* spp. infections [158,246]. *Aspergillus fumigatus* has developed mechanisms of resistance to azoles through either long-term exposure to low concentrations of azoles administered to human subjects or exposure to environmental organic material which is rich in aspergilli and contains traces of agricultural azoles [246,247,248]. The effect of fluconazole on resistance appearance is of special interest due to its common use as a prophylactic treatment. A meta-analysis of placebo-controlled RCTs in preterm infants showed that fluconazole prophylaxis has higher effectiveness and a comparable incidence of side effects and resistance compared to the placebo [133].

### 2.8. Future Perspectives Regarding Antibiotic Use in Neonates

Overall, the data presented above show that there are still unresolved issues regarding antibiotic use in neonates. Among the most crucial problems are the following: (a) insufficient PK data and RCTs resulting in the off-label use of most antibiotics in neonates and the variation of dosing regimens between centers; (b) the almost completely lacking data on the long-term effects of early antibiotic exposure on infants’ ND, which outline the urgent need for long-term follow-up studies focusing on the ND of children exposed to antibiotics before or soon after birth; and (c) the increasing incidence of antimicrobial and antifungal drug resistance of pathogens, which may not only have detrimental effects on patient outcomes but also constitutes a global public health problem. The first step to reducing the emergence of antibiotic-resistant strains is the rapid identification of pathogens’ resistance to antibiotics and their appropriate management. In addition, research is focusing on the discovery of new antibiotics that are currently in clinical trials to be evaluated for their antibacterial–antifungal activity, effectiveness, and safety and the potential of reversing previously resistant phenotypes [249,250]. The development of antifungal vaccines against fungal cell surface proteins in human subjects using mRNA technology seems to be an attractive option to combat antifungal drug resistance development [251]. Another research target that is currently under investigation is the discovery of medications already in use for indications other than antibacterial–antifungal ones, such as statins, to be used as adjuncts to prevent or reverse resistance. In this respect, there is evidence that statins may act against fungal infections in synergy with antibiotics [252,253]. Moreover, the emergence of nanotechnology-based drug delivery systems, which have altered dramatically traditional antibiotic treatments, may present new opportunities to enhance bacterial susceptibility and overcome bacterial resistance due to biofilm formation [254]. Finally, another promising alternative is the use of antimicrobial and antifungal peptides, which are natural peptides exerting broad-spectrum antimicrobial and anti-*Candida* species activity. These properties make them potential candidates for the fight against neonatal infections, after modification to enhance their stability, bioavailability, and therapeutic potential [255].

## 3. Analgesics and Sedatives

During the last few decades, it has become evident that fetuses and preterm infants not only feel pain, but are more sensitive and show cardio-respiratory, hormonal, and metabolic stress responses similar to, or even more intense than, that seen in adults [256]. This is of great importance as neonates receiving intensive care are exposed to numerous painful and/or stressful procedures [257]. Moreover, cumulative evidence suggests that prolonged exposure to painful events in the neonatal period is associated with significant long-term consequences [258,259]. In this context, international scientific societies have provided guidelines for the prevention and management of pain and stress in neonates [260,261,262].

### 3.1. Analgesic Drugs

#### 3.1.1. Opioids

The opioids morphine, fentanyl, and remifentanil are the most frequently employed analgesic agents in neonates [4,8]. Typically, they are used for procedural or post-operative pain, either as monotherapy or in combination with other drugs [263,264]. Overall, the use of specific pain scales in neonates has revealed that opioids reduce procedural pain. However, there is considerable uncertainty regarding the relationship between opioids and episodes of bradycardia, hypotension, or severe apneas [265]. Therefore, the use of standardized protocols for pain management have been suggested to minimize their exposure to opioids [262,266].

##### Morphine

Morphine is a commonly used medication for analgesia in neonates. It has a slow onset of action (mean onset in 5 min), reaches its peak effect in 15 min, and is metabolized in the liver via glucuronidation, oxidation, and sulfidation [264]. The glucuronide byproducts of morphine include morphine-3-glucuronide and morphine-6-glucuronide. The latter metabolite has a strong affinity with the morphine receptor and thereby possesses analgesic properties and augments the analgesic effect of morphine [267,268]. The main indication for morphine analgesia is invasive mechanical ventilation in preterm neonates, while it is not recommended for procedural pain in non-ventilated neonates, such as examinations for retinopathy of prematurity [269,270].

Variable dosing regimens have been recommended by various authors and scientific societies. Early studies by Quinn et al. and Chay et al., based on available PK studies, recommended morphine dosing regimens with loading doses of 100 and 150 mcg/kg/h, respectively, for about 2 h followed by a continuous IV infusion of 25 or 22.5 mcg/kg/h, respectively [271,272]. Based on these studies, Saarenmaa et al. administered morphine in ventilated neonates at a loading dose of 140 mcg/kg over one hour, followed by a continuous IV infusion of 20 mcg/kg/h for at least 24 h [268,272]. Five years later, Anand et al., in a study of 898 ventilated neonates from 16 centers, administered morphine at a dosing regimen based on the PK studies available at the time of the study [269]. The treatment protocol included a morphine loading dose of 100 mcg/kg in an IV infusion over 1 h, followed by continuous infusions ranging from 10 to 30 mcg/kg/h depending on GA [269]. No increase in early neurological adverse effects was associated with morphine use, except for in hypotensive infants and ELBWIs who received high doses (>10 mcg/kg/h) [269].

The reported acute adverse effects include respiratory depression, miosis, hypotension, constipation, increased biliary pressure, urinary retention, tolerance, and withdrawal [264,269]. The results of the NOPAIN pilot trial suggested that morphine administered prophylactically in ventilated preterm infants may improve neurologic outcomes [273]. However, as documented in a subsequent RCT (NEOPAIN), pre-emptive analgesia with morphine did not decrease the composite outcome of death, severe intraventricular hemorrhage, or periventricular leukomalacia in preterm neonates, while intermittent boluses of morphine actually increased the incidence of the composite outcome [269]. Overall, the results of these studies in preterm infants warrant extreme caution in the use of morphine during key stages of brain development, especially in ELBWIs. It should be noted, though, that it is difficult to solely attribute these adverse outcomes to morphine (and other opioids), as a low GA and pre-existing arterial hypotension are also associated with intraventricular and periventricular hemorrhage [259,264,274,275,276].

Interestingly, the analgesic effectiveness of morphine in preterm neonates was questioned as well. The results of the Procedural Pain in Premature Infants (POPPI) study showed no beneficial effect of morphine on procedural pain in preterm neonates, whereas a higher number of morphine-treated neonates required non-invasive ventilation due to apneas compared to the placebo group [270].

##### Fentanyl

Fentanyl is a synthetic opioid characterized by a rapid onset of action, within 1–2 min when administered intravenously, and an intermediate duration of action (30 min) [277]. These properties have made this medication a suitable opioid for acute, short-lasting procedural pain [264,268]. A RCT comparing fentanyl to morphine as an analgesia for ventilated neonates showed their equal efficacy, with fentanyl having fewer side effects [268]. As a result, fentanyl has become the most commonly used analgesic sedative medication in many NICUs [4,264,268].

PK studies on fentanyl use in neonates and children are scarce. A comprehensive review by Ziesenitz et al. showed significant age-related changes and a great variability in fentanyl kinetics in preterm infants following bolus or continuous intravenous infusion, with a clearance ranging widely between 3.4 and 58.7 mL/min/kg [278]. The dosing regimens used in studies in neonates have included loading doses ranging from 5 to 12.5 mcg/kg followed by a continuous infusion of 0.5 to 2.0 mcg/kg/h [279]. A recent PK study indicates a quickly increasing clearance threshold within the first three postnatal weeks in preterm infants, allowing for a reduced infusion dose by 50% and 25% on postnatal days 0–4 and 5–9, respectively. These results support a decrease in the fentanyl dose regimen potentially mitigating some of the adverse effects of fentanyl [280]. Schofer et al. suggested a dose of 3 mcg/kg of fentanyl IV three minutes prior to intubation. Doses greater than 5 mcg/kg have been associated with an increased incidence of hypotension [281].

The serious adverse effects of fentanyl include dose-dependent respiratory depression, chest wall rigidity, and arterial hypotension [281]. Of note, in a retrospective study involving very low GA infants, fentanyl was independently associated with the need for inotropes [274]. Nevertheless, the effect of fentanyl on blood pressure in neonates is still unclear. A recent Cochrane review including 13 independent studies (enrolling 823 newborn infants) concluded that opioids probably are more effective in reducing pain scores than a placebo. However, existing evidence cannot clarify the effect of opioids on episodes of bradycardia, hypotension, or apnea [265,278]. Thus, no definite conclusion could be reached concerning the potential effect of opioids, in general, or fentanyl, per se, on blood pressure in preterm and term infants [265,278]. A long-term study of infants treated with fentanyl soon after birth did not find any significant correlation between cumulative fentanyl exposure and ND outcomes at five years of age [282]. These results were consistent with previous reports on VLBWIs at the age of two years [283].

##### Remifentanil

Remifentanil is another synthetic opioid and a selective morphine-receptor agonist with rapid onset and an ultra-short duration of action due to its quick degradation by nonspecific plasma and tissue esterases [284]. In neonatology, it has been used for brief procedural analgesia, i.e., prior to intubation, and prolonged sedation/analgesia with relative safety [264,285,286].

When using remifentanil (1 to 3 mcg/kg) as a single premedication for INSURE, a faster infusion over 30 s (compared to over 60 s) was associated with a higher incidence of chest rigidity (43%) and a shorter duration of sedation [287]. Due to the risk of chest wall rigidity, it should only be used in intensive care units with strict monitoring capabilities. The long-term consequences of remifentanil administration in neonates are unknown and, therefore, its optimal use in the neonatal population warrants further studies [264,284,286,287,288,289].

#### 3.1.2. Non-Opioid Analgesics

In the context of the existing controversy surrounding the effectiveness and safety of opioids, alternative analgesics, mainly paracetamol (acetaminophen), have been used for the treatment of postoperative and procedural pain in neonates. It exerts its central analgesic effect via the activation of descending serotonergic pathways and the inhibition of prostaglandin synthesis [290].

Pooled analyses of PK results suggest an oral dose of 25 mg/kg/day in preterm neonates at 30 weeks, 45 mg/kg/day at 34 weeks, 60 mg/kg/day in term neonates, and 90 mg/kg/day at 6 months of age [291,292]. Other authors recommend a loading dose of 20 mg/kg, followed by 10 mg/kg every 6 h, for 32–44 week-old neonates. For neonates < 32 weeks old, a loading dose of 12 mg/kg and a maintenance dose of 6 mg/kg every 6 h is recommended [293].

The safety profile of paracetamol might have contributed to its increasing use in term and preterm neonates, despite its off-label use in this population [291,294]. A recent meta-analysis and a review showed that existing data are not sufficient to support the role of paracetamol in reducing procedural pain in neonates but that it may reduce the need for morphine following major surgery [290,295].

### 3.2. Sedatives

#### 3.2.1. Benzodiazepines

Benzodiazepines effectively relieve patients’ stress, but they exert no analgesic action. Therefore, they are mainly used as an adjunct to analgesics and may rarely be used for minor procedures [296]. Midazolam is the most common sedative utilized by NICU physicians for sedation [4,8]. Due to its pharmacological advantages (a lack of active metabolites), it has replaced diazepam [297]. Midazolam is a short-acting benzodiazepine that possesses sedative and anticonvulsant properties [296]. The advantages of midazolam over other sedatives are its rapid onset of action and the fast termination of its effects [298].

Various dosing regimens have been recommended, but one report suggested that when midazolam is used as the only sedative agent, the optimal dose was 209 mcg/kg/h (range: 100 to 500 mcg/kg/h) [296]. However, the limited existing data raise significant concerns about its safety when given as a continuous infusion for sedation in infants receiving intensive care [296].

The adverse effects reported in neonates include respiratory depression, hypotension, and a decrease in cerebral blood flow. Moreover, paradoxical agitation, such as hyperexcitability and myoclonus, have been reported, which can be attributed to the low number of gamma-amino-butyric acid (GABA)-A receptors in neonates [299]. As documented in the most recent Cochrane review (2017) of this drug, which included only three trials and 148 neonates, the duration of the NICU stay was significantly longer in the midazolam group than in the placebo group. Moreover, in one of the included studies, the incidence of a combined adverse outcome of death, grade 3 or 4 peri-intraventricular hemorrhage, or periventricular leukomalacia at a PNA of 28 days was significantly higher in the midazolam group compared to the morphine group [273,300]. Consequently, the limited existing data raise significant concerns about its safety when given as a continuous infusion for sedation in infants receiving intensive care.

#### 3.2.2. Ketamine

Ketamine is an analgesic and anesthetic (analgo-sedative) agent that has been used more in children than in neonates. It is an antagonist of n-methyl D-aspartate (NMDA) receptors, gamma-aminobutyric acid (GABA), and other brain receptors, and it acts rapidly [301]. It is indicated as analgesia for minor procedures in neonates, such as cannulation for extracorporeal membrane oxygenation [302]. It has also been administered for endotracheal suctioning at a dose of 2 mg/kg [302].

Several administration routes have been used (intravenous, intramuscular, or intranasal). Its recommended analgesic doses are 0.15–0.25 mg/kg IV or 0.5–1 mg/kg intramuscularly [301]. Overall, it is believed to offer cardiorespiratory stability, as ketamine causes mild increases in BP and heart rate while having minimal effects on cerebral blood flow. Moreover, it decreases respiratory drive and induces bronchodilation [302].

Few perioperative complications and satisfactory operative conditions were reported with ketamine analgesia compared to general anesthesia during laser treatments of retinopathy of prematurity in a small group of infants [303]. In the larger “NOPAIN-ROP” RCT, IV fentanyl and IV ketamine were tested for their pain relief during laser photocoagulation for retinopathy of prematurity in preterm infants. With both drug regimens, adequate analgesia was provided only in a minority of infants [304]. Concerns regarding potential ketamine-mediated neurotoxicity in the immature brain give us pause regarding the use of ketamine infusion as a therapeutic option for refractory neonatal seizures [305].

#### 3.2.3. Propofol

Propofol is a highly lipophilic compound that is rapidly distributed from the blood to the subcutaneous fat and the CNS, with subsequent redistribution and metabolic clearance. It positively modulates the inhibitory function of the neurotransmitter GABA through ligand-gated GABA-A receptors. It is a short-acting sedative (without analgesic properties) that is rapid in its onset. These properties make propofol attractive for short-duration interventions. On the other hand, due to its reduced clearance capacity, both preterm and term neonates in their first week of postnatal life are at risk for accumulation following propofol administration [306].

In a recent prospective trial investigating the optimal propofol dose in neonates requiring non-emergency endotracheal intubation, effective sedation without side effects was reported as “difficult to achieve”; an optimal result was observed more often with the high starting dose of 2.0 mg/kg compared to the lower doses of 1.0 and 1.5 mg/kg. However, propofol-induced hypotension occurred in 59% of patients, and this risk was found even with low initial doses [307]. In a subsequent study, the profound and prolonged decrease in blood pressure following a propofol administration as premedication for intubation in neonates was mainly attributed to its starting dose rather than the cumulative dose [308]. There is only one Cochrane review of propofol for procedural sedation/anesthesia in neonates and it was published more than a decade ago. No practice recommendations could be made at that time [309]. Additionally, there has been no updated review, which reflects the lack of available evidence regarding the use of propofol in neonates.

#### 3.2.4. Alpha-2 Agonists

Centrally acting alpha-2 agonists, such as clonidine and dexmedetomidine, possess sedative, analgesic, and anxiolytic properties. The two main adverse effects of alpha-2 agonists are bradycardia and hypotension. However, contrary to opioids, they do not cause significant respiratory depression. Due to this advantage, alpha-2 agonists have been used in critically ill children as adjunctive sedative agents alongside opioids and benzodiazepines and help to minimize the use of benzodiazepines and opioids in children and prevent withdrawal syndrome [310]. There are limited data on the “off-label” use of clonidine and dexmedetomidine in neonates.

The most recent (2017) relevant Cochrane review included only one small trial (112 neonates) comparing clonidine with a placebo. Although sedation–pain scale values were lower among treated infants, clonidine was not associated with reduced death, duration of mechanical ventilation, or duration of their stay in the NICU [311]. Interestingly, there are no studies regarding the use of clonidine for the prevention or treatment of procedural and postoperative pain, or pain associated with clinical conditions in non-ventilated neonates [312]. Clonidine has been used for neonatal abstinence syndrome, as well. In a systematic review of RCTs, clonidine was found to be more efficacious than morphine with respect to the duration of treatment, and better than phenobarbital in reducing morphine treatment days [313]. The potential adverse effects of clonidine include hypotension, rebound hypertension, bradycardia, syndrome of inappropriate antidiuretic hormone, and postoperative apnea. At high doses, it can probably cause respiratory depression.

Like clonidine, dexmedetomidine was reported to be effective in achieving sedation/analgesia in neonates and reducing the need for adjunctive sedative or analgesic agents. Furthermore, it was found to decrease the time to extubation and the duration of mechanical ventilation [314]. It is worth noting that, over the last several years, dexmedetomidine has attracted the interest of neonatologists as an analgesic/sedative agent in neonates undergoing therapeutic hypothermia due to its possible neuroprotective effect [315,316]. However, so far, there are insufficient data from RCTs evaluating the use of any analgesic sedative agent during therapeutic hypothermia, including clonidine and dexmedetomidine [317]. Dexmedetomidine has been used in neonates at a starting dose of 0.2 to 0.3 mcg/kg/h, escalating in 0.1 mcg/kg/h increments, depending on sedation–pain assessment scores, up to a median maximum dose of 0.5 mcg/kg/h (Table 4 and Table 5) [318,319]. The main side effects of dexmedetomidine are bradycardia and hypotension.

The suggested dosing regimens of the analgesics and sedatives which have been most frequently used in neonates are summarized in Table 4. An executive summary regarding selective analgesic/sedative drug data is given in Table 5.

## 4. Antiseizure Drugs

### 4.1. General Considerations for Antiseizure Drugs

Antiseizure medications (ASMs) are used to treat seizures, to which the neonatal brain is prone. Currently, hypoxic–ischemic encephalopathy, stroke, brain infections, intracranial hemorrhage, cerebral dysplasias, hypoglycemia, electrolyte disturbances, epileptic encephalopathies, inborn errors of metabolism, and benign familial neonatal epilepsy are the main causes of neonatal seizures [320,321]. Nevertheless, thus far, there is limited evidence regarding the best pharmacological treatment for neonatal seizures. This fact explains the existing variability among centers and neonatologists as to whether, when, and for how long ASMs should be used, as well as which medication should be chosen [322]. Additionally, there are concerns related to their side effects, drug interactions, the monitoring of blood levels, and long-term ND effects [322].

In general, the choice of the optimal ASM takes into consideration the etiology and severity of the seizures, but also the cardiac, renal, and liver function of the neonate. ASMs exert their actions via GABA receptors (i.e., phenobarbital), sodium channels (i.e., phenytoin, carbamazepine), by binding to synaptic vesicle protein SV2a (levetiracetam), or via the N-methyl-D-aspartate [NMDA] receptor as a glutamate antagonist (ketamine) [321]. In contrast to adults and older children, only very few ASMs have been licensed for use in infants and neonates [308,323,324].

Herein, we will briefly refer to phenobarbital, levetiracetam, and phenytoin/fosphenytoin, which are the most commonly used ASMs for the treatment of clinically suspected and/or seizures confirmed by electroencephalography in neonates (Table 6).

### 4.2. Antiseizure Drug Characteristics

#### 4.2.1. Phenobarbital

Phenobarbital (or phenobarbitone) is a barbiturate with antiseizure and hypnotic/sedative properties. Phenobarbital sodium (a powder for injection) is the first and only medication approved by the FDA for the treatment of neonatal seizures [325]. Phenobarbital acts by increasing the GABA-mediated inhibition of GABA [325]. The drug is metabolized in the liver and excreted in the urine. Regardless of seizure etiology, phenobarbital is the most frequently used ASM in the NICU setting [320].

According to the recent recommendations by The Neonatal Task Force of the International League Against Epilepsy (ILAE), phenobarbital should be the first-line ASM (an evidence-based recommendation) regardless of etiology (with expert agreement), unless channelopathy is likely the cause of seizures (e.g., due to family history), in which case phenytoin or carbamazepine should be used. Phenobarbital should be given at a loading dose of 20 mg/kg IV, followed by a maintenance dose of 5 mg/kg/day IV or orally. A second loading dose at 10–20 mg/kg could be administered IV if required [324]. A 40% response was reported after the initial loading dose, but the sequential administration of IV phenobarbital led to the improved control of seizures in term and preterm neonates [325]. Based on the results of a retrospective population PK study across a pediatric population including neonates, an initial phenobarbital dose of 30 mg/kg has the highest probability of attaining a therapeutic concentration at seven days. Moreover, PMA and drug–drug interactions should be incorporated into dosing regimens [326]. The monitoring of circulating levels (target concentration: 20–40 mcg/mL) should be considered if the neonate is on maintenance therapy [324,326]. In asphyxiated neonates with seizures who are undergoing therapeutic hypothermia, a second loading dose of 20 mg/kg has been suggested for better seizure control. Nevertheless, the prophylactic administration of phenobarbital before the onset of hypothermia is not recommended [327].

The most common adverse effects of phenobarbital include respiratory depression, hypotension, depressed consciousness, somnolence, and poor feeding [320,325]. Moreover, the fact that phenobarbital may cause apoptotic neurodegeneration in the developing rat brain at plasma concentrations relevant for seizure control in humans is alarming [328].

#### 4.2.2. Phenytoin/Fosphenytoin

Phenytoin and fosphenytoin (the phosphorylated prodrug of phenytoin) [329] are still considered second-line ASMs for most seizure etiologies in neonates not responding to phenobarbital [324,329]. However, their use has declined during the last two decades and has been surpassed by levetiracetam, which is now the second most widely used antiseizure medication in NICUs [330].

With respect to seizure control, two studies evaluating phenytoin and fosphenytoin in neonates demonstrated similar efficacy to phenobarbital (45% and 56% became seizure-free after an initial treatment with phenytoin and fosphenytoin, respectively) [331,332]. Ιn a recent meta-analysis, high uncertainty was expressed about the effect of phenobarbital compared to phenytoin in achieving seizure control after the maximum loading dose of each ASM [333].

A loading dose of phenytoin/fosphenytoin (20 mg/kg phenytoin-equivalent IV over 30 min) is initially administered, followed by maintenance (5 mg/kg/day IV or orally in two divided doses). Adjustments are made according to the response (max. per dose 7.5 mg/kg) and its plasma concentration (target level: 10–20 mcg/mL). Phenytoin has poor oral bioavailability and limited hepatic metabolism capacity. Low albumin levels and bilirubin displacement by phenytoin from its protein-binding sites may result in increased serum levels of free bilirubin. Its levels may also increase in infants receiving therapeutic hypothermia [324].

Careful cardiac monitoring is needed during and after administering IV phenytoin/fosphenytoin because of the risk of severe hypotension and cardiac arrhythmias. Other common adverse reactions are infection, site irritability/necrosis, hypotonia, and respiratory depression/arrest [324]. Fosphenytoin is preferred over phenytoin due to the lower risk of adverse effects [329]. Similar to phenobarbital, there are experimental data indicative of apoptotic neurodegeneration with phenytoin [328].

#### 4.2.3. Levetiracetam

Levetiracetam has been an FDA-approved ASM for adults and infants older than a one-month PNA since 2012, and it is mainly used in combination with other ASMs. According to a prospective cohort study, it is used off-label in neonates, as it is the next most commonly used ASM for neonatal seizures after phenobarbital [320]. A reduction by 50–88% in the frequency of seizures without serious side effects has been reported using levetiracetam [334,335]. Moreover, when used as a first-line ASM drug for neonatal seizures, levetiracetam achieved better control than phenobarbital [336]. However, in another recent phase IIb study, phenobarbital was found to be more effective than levetiracetam for the treatment of neonatal seizures (80% versus 26%, respectively), although more adverse effects were observed in subjects assigned phenobarbital [337]. This finding is also supported by a recent (2023) Cochrane meta-analysis; it was concluded that phenobarbital as a first-line ASM is probably more effective than levetiracetam in achieving seizure control after the first loading dose and after the maximum loading dose (moderate certainty of evidence) [333]. It should be noted that levetiracetam might have a neuroprotective effect on the neonatal brain by reducing neuronal apoptosis, as shown in animal models [338].

The ILAE suggested that levetiracetam should be administered as follows: a loading dose of 40 mg/kg IV, followed by a second loading dose of 20 mg/kg IV if required, and a maintenance dose of 40–60 mg/kg/day IV or orally in three divided doses [324]. However, higher loading doses (60 mg/kg) may be more effective and substantially decrease the seizure burden [337]. Moreover, high doses seem to be well tolerated. Specifically, in a neonatal study, the initial cumulative dose of levetiracetam ranged from 50 to 100 mg/kg [339], while, in another study involving infants suffering hypoxic–ischemic encephalopathy, the mean total and maintenance doses of levetiracetam were 63 and 65 mg/kg/day, respectively [340]. In clinical practice, the transition from IV to oral administration should be carried out, when feasible, and at equivalent doses. Levetiracetam is mainly excreted from the kidneys and, to a lesser degree, from the liver, resulting in fewer interactions with other drugs [321,337].

The reported side effects of levetiracetam include mild sedation, irritability, and increased blood pressure [321,337].

#### 4.2.4. Midazolam

The use of midazolam as a sedative in ventilated infants is discussed elsewhere in this article. Midazolam was approved in 2022 by the FDA for rescue treatment in adults with status epilepticus, but not in infants or neonates. However, this non-FDA approved benzodiazepine has been utilized for the management of refractory neonatal seizures as well [296,298,341].

Castro-Conde et al. reported that midazolam effectively controlled EEG-confirmed seizures in all non-responders (*n* = 13) to phenobarbital/phenytoin, significantly improving their long-term ND [341]. The authors proposed an antiseizure dosing regimen comprising a 0.15 mcg/kg IV bolus, followed by a continuous infusion of 1 mcg/kg/min, increasing by 0.5 to 1 mcg/kg every 2 min, up to a maximum of 18 mcg/kg/min. In other studies, a lower efficacy was reported [342,343]. In the recommended treatments made by the ILAE for the management of neonatal seizures, midazolam is included in the second-line ASM options. Its administration includes a loading dose of 0.05–0.15 mg/kg, followed by the continuous infusion of a maintenance dose (1 mcg/kg/min), which may be titrated up in steps of 1 mcg/kg/min to a *max*. of 5 mcg/kg/min) [324].

Midazolam may cause respiratory depression, hypotension, and poor feeding, as well as dyskinetic movements and myoclonus [300]. Nevertheless, most worrisome is its potential effect on brain development and ultimately ND. Animal data showed that benzodiazepines may induce the apoptotic neurodegeneration of the developing brain [328], although these concerns have not been validated in human studies [344].

#### 4.2.5. Topiramate

Topiramate, an anticonvulsant agent widely used in adults and children, is characterized by good absorption, high bioavailability, and good tolerability [345]. Due to the encouraging experimental evidence of it working as a neuroprotective agent (through several mechanisms including glutamate-receptor inhibition), topiramate has received additional interest as an adjunct treatment in neonates undergoing therapeutic hypothermia [346]. In the “NeoNATI” feasibility study, 21 neonates were allocated to hypothermia plus topiramate at a dose of 10 mg/kg once daily for the first three days of life, and 23 to hypothermia alone. The co-administration of topiramate was safe and associated with a reduction in the prevalence of epilepsy, while it did not reduce the frequency of the combined outcome of mortality or severe neurological disability [347].

Investigators also evaluated the influence of hypothermia on topiramate PK. In an early study by Filippi et al. (2009), 13 neonates undergoing whole-body hypothermia were given oral topiramate (5 mg/kg once a day for the first three days of life) while 7 received a concomitant phenobarbital treatment. In most neonates (11/13), topiramate concentrations were within the reference range for the entire treatment duration [348]. A therapeutic range of 5–20 mg/L has been proposed for epilepsy therapy [349,350]. A more recent study evaluated topiramate PK in hypothermic neonates. Aiming to decrease seizure events, topiramate was given at 5 mg/kg on day 1 and at 3 mg/kg on days 2–5. Based on its PK, the authors suggested an optimized alternative dosage regimen consisting of a loading dose of 15 mg/kg for cycle one and maintenance doses of 5 mg/kg for the following four cycles to allow topiramate concentrations to be within the therapeutic range after the first dose in more than 90% of cooled neonates [350,351].

Topiramate is well tolerated. No adverse effects on respiratory and hemodynamic parameters or hematological and biochemical tests have been reported [347].

**Table 6:** Selective antiseizure medications most often used in neonates.

Medication [References]	Mechanisms of Action	Main Indications	Dosing Regimen	Side Effects
Phenobarbital [320,324,325,327]	Increases GABA-A-mediated inhibition of GABA.	First-line ASM regardless of seizure etiology.	LD: 20 mg/kg, IV, (up to a total dose of 40 mg/kg); MD: 5 mg/kg/day, IV or orally, in one dose.	Hypotension, poor feeding, sedation, respiratory depression, bradycardia, and hepatotoxicity.
Phenytoin/fosphenytoin [324,329]	Sodium channel blocker.	Second-line ASM.	LD: 20 mg/kg, IV; MD: 5–7.5 mg/kg/day, IV or orally, in two doses.	Hypotension, cardiac arrhythmias, irritability/necrosis, hypotonia, and respiratory depression/arrest.
Levetiracetam [321,324,336]	Binds to synaptic vesicle protein SV2a	Second-line ASM; maybe first-line in some NICUs.	LD: 40 mg/kg/day, IV, up to a total dose of 60 mg/kg; MD: 40–60 mg/kg/day, IV or orally, in 3 doses.	Mild sedation, irritability, and increased blood pressure.
Midazolam [296,298,300,341,351]	Binds to GABA-A receptors.	Refractory neonatal seizures.	LD: 0.05–0.15 mg/kg IV, MD: 1–5 mcg/kg/min, continuous IV infusion, titrated up in steps of 1 mcg/kg/min to a *max*. of 5 mcg/kg/min.	Respiratory depression, hypotension, poor feeding, dyskinetic movements, and myoclonus
Topiramate [346,348,349,350]	Inhibition of glutamate receptors.	Antiepileptic, potentially neuroprotective for HIE; Mainly therapeutic hypothermia.	Optimized dosing regimen LD: dose of 15 mg/kg for cycle one; MD: 5 mg/kg for the following four cycles.	No adverse effects on respiratory and hemodynamic parameters or hematological and biochemical tests.

ASMs, antiseizure medications; GABA, gamma-amino-butyric acid; HIE, hypoxic–ischemic encephalopathy; IV, intravenously; LD, loading dose; MD, maintenance dose; max, maximum; NICU, neonatal intensive care unit.

An executive summary of selective antiseizure drugs is shown in Table 7.

## 5. Future Perspectives and Conclusions

In clinical practice, neonatologists use several drug categories, often with very little proof of their safety and efficacy. This fact is indicative of important knowledge gaps in neonatal pharmacology and the need for new treatments. Therefore, there is an urgent need for clinical trials in which the diversity of neonates and their medical conditions will be taken into consideration. Only then will we be able to identify the most effective (old or new) medication and, at the same time, decrease the risk of adverse effects, especially concerning the developing brain, thus improving ND outcomes. Additionally, scientific information derived from the application of new technologies (e.g., genetics, -omics, nanotechnology), along with the analysis of pharmacometric parameters and clinical-imaging data, may allow for a more precise use of medications in the neonatal population. Although improvements have been made in legislation, infrastructure, and clinical trial methodologies during the last decades, ensuring a safer framework surrounding neonatal drug development, more work is still needed [352].

## Figures and Tables

**Table 4:** Analgesics and sedatives that are commonly used in neonates.

Medication [References]	Mechanisms of Action	Main Indications	Dosing Regimen	Side Effects
**Analgesic drugs**				
Morphine [259,268,269,270,271,272]	Opiate receptor agonist.	Pre-emptive analgesia in intubated and ventilated preterm neonates.	ID: 100–150 mcg/kg/h, IV; MD: 20–30 mcg/kg/h, IV, for =/>24 h. Lower doses may be needed in liver and renal dysfunction.	Respiratory depression, miosis, hypotension, constipation, increased biliary pressure, urinary retention, and tolerance and withdrawal syndrome.
Fentanyl [263,264,268,278,279,281]	Opiate receptor agonist.	Acute painful procedures, such as intubation.	LD: 5 to 12.5 mcg/kg, IV, followed by infusion of 0.5 to 2.0 mcg/kg/h, IV. Doses > 5 mcg/kg were associated with increased incidence of hypotension.	Respiratory depression, chest wall rigidity, and hypotension. No association with long-term neurodevelopment.
Remifentanyl [286,287,288,289]	Opiate receptor agonist.	Premedication prior to intubation. Procedures and surgeries of short duration.	Fast bolus of 1–3 mcg/kg IV within 60 s.	Hypotension and chest wall rigidity. Insufficient sedation and severe side effects after fast adm.
Acetaminophen [264,291,292,293,294,295,319]	Activation of descending serotonergic inhibitory pathways.	Mild to moderate procedural or postoperative pain. Adjunctive therapy to opioids in moderate to severe pain; reduces the use of opioids. FDA approval for >2 years.	Oral or rectal adm: 25–60 mg/kg/day, depending on GA. IV adm: 20–40 mg/kg/day depending on GA.	Hepatotoxicity, bradycardia, and hypotension.
**Sedatives**				
Midazolam [273,296,299,300,302]	Induces the inhibitory function of GABA through GABA-A receptors.	Adjunct to analgesics; rarely used alone in minor procedures.	Dosing for sedation: 209 mcg/kg/h (range: 100 to 500 mcg/kg/h) IV.	Respiratory depression with hypotension, decrease in CBF, agitation (hyperexcitability and myoclonus).
Ketamine [301,302,304,305]	NMDA receptor and other brain receptor antagonist.	Minor procedures (i.e., intubation, endotracheal suctioning, cannulation for ECMO).	Analgesic doses: 0.15–0.25 mg/kg, IV or 0.5–1 mg/kg intramuscularly. Endotracheal suctioning: 2 mg/kg, IV.	Mild increase in blood pressure and heart rate, minimal effects on CBF, suppresses respiratory drive, and bronchodilation.
Propofol [306,308,309]	Induces the inhibitory function of GABA through GABA—A receptors.	Short-duration interventions.	High ID (2.0 mg/kg) produces better results than lower doses (1.0 and 1.5 mg/kg).	Profound hypotension, especially with high dose. No practice recommendations.
α2-Agonists (clonidine; dexmedetomidine) [310,311,313,314,315,317,318]	Centrally acting alpha-2 agonists.	Adjunctive to opioids and benzodiazepines, reducing their use. Therapeutic hypothermia Neonatal abstinence syndrome. Post-operatively after major surgeries.	Clonidine: 6 mcg/kg/d, titrated up to 9 mcg/kg/d. Dexmedetomidine: ID: 0.2 to 0.3 mcg/ kg/h titrated up in 0.1 mcg/kg/h increments as required.	Clonidine: Hypotension, rebound hypertension, bradycardia, syndrome of inappropriate antidiuretic hormone, and postoperative apnea. At high anesthesia, probably respiratory depression. Dexmedetomidine: bradycardia and hypotension.

adm., administration; CBF, cerebral blood flow; ECMO, extracorporeal membrane oxygenation; FDA, Food and Drug Administration; GA, gestational age; GABA, gamma-amino-butyric acid; h, hour; ID, initial dose; IV, intravenous; LD, loading dose; NMDA, n-methyl D-aspartate.

**Table 5:** Executive summary of selective analgesic/sedative drug data. Dose regimens are shown in Table 4.

The opioids morphine, fentanyl, and remifentanil are the most frequently employed analgesic agents in neonates [4,8]. The main indication for morphine analgesia is invasive mechanical ventilation in preterm neonates [269,270]. Pre-emptive analgesia with morphine did not improve the composite outcome of death and neurodevelopmental impairment in preterm neonates, while intermittent boluses of morphine increased the incidence of the composite outcome [269]. Fentanyl is characterized by a rapid onset of action (in 1–2 min when administered intravenously) and a 30 min duration of action [277]. Serious adverse effects include dose-dependent respiratory depression, chest wall rigidity, and arterial hypotension [281]. Early fentanyl exposure is not correlated with neurodevelopmental outcomes at five years of age [282]. Remifentanil has been used mainly for brief procedural analgesia, i.e., prior to intubation [264,285,286]. Paracetamol (acetaminophen) has been used for the treatment of postoperative and procedural pain in neonates. Midazolam has a rapid onset of action and fast termination of effects [298]. Ketamine is indicated as analgesia for minor procedures in neonates [301] and for endotracheal suctioning [302]. There are concerns regarding potential ketamine-mediated neurotoxicity in the immature brain [305]. Propofol has a rapid onset and short duration and therefore is used for short-duration interventions. There is a lack of available evidence regarding the use of propofol in neonates. alpha-2 agonists, clonidine and dexmedetomidine, have been used in critically ill children as adjunctive sedative agents alongside opioids and benzodiazepines [310].

**Table 7:** Executive summary of selective antiseizure drug data. Dose regimens are shown in Table 7.

Phenobarbital, levetiracetam, and phenytoin/fosphenytoin are the most often used ASMs for the treatment of clinically suspected and/or confirmed seizures. Phenobarbital is the first-line ASM regardless of etiology, unless channelopathy is likely the cause of seizures, in which case phenytoin or carbamazepine should be used [324]. Phenobarbital’s most common adverse effects include respiratory depression, hypotension, depressed consciousness, somnolence, and poor feeding [320,325], while its association with apoptotic neurodegeneration in the developing rat brain is a major concern [328]. Phenytoin and fosphenytoin constitute second-line ASMs for most seizure etiologies in neonates not responding to phenobarbital [324,329]. Phenytoin and fosphenytoin’s main adverse effects are severe hypotension and cardiac arrhythmias. Other common adverse reactions are infection, site irritability/necrosis, hypotonia, and respiratory depression/arrest [324]. There are concerns about apoptotic neurodegeneration with phenytoin [328]. Levetiracetam is the next most used ASM for neonatal seizures following phenobarbital [320]. High doses are well tolerated [337,338]. Midazolam has been used for refractory neonatal seizures [298,341]. Topiramate is an anticonvulsant agent with good tolerability [345] and neuroprotective actions, supporting its use as an adjunct treatment in neonates undergoing therapeutic hypothermia [346]. Well tolerated.

ASMs, antiseizure medications.

## Data Availability

Not applicable.

## Supplementary Materials

The following supporting information can be downloaded at https://www.mdpi.com/article/10.3390/children11070871/s1, Table S1. Detailed presentation of selective antimicrobial drugs commonly used in neonates. Table S2. Detailed presentation of selective antifungal drugs commonly used in neonates.

## Abbreviations

5FC	5-fluorocytosine
5FU	5-fluorouracil
AmB-D	amphotericin Β deoxycholate
ASMs	antiseizure medications
BW	birth weight
*C.*	*Candida*
CNS	central nervous system
CoNS	*coagulase-negative Staphylococci*
CS	cesarean section
CSF	cerebrospinal fluid
*E.*	*Escherichia*
ELBWIs	extremely low birth weight infants
EOS	early-onset sepsis
ESBLs	extended-spectrum β-lactamases
FDA	Food and Drug Administration
GA	gestational age
GABA	gamma-amino-butyric acid
GBS	Group B Streptococcus
IBD	inflammatory bowel disease
IC	invasive candidiasis
ILAE	International League Against Epilepsy
IV	intravenously
LAmB	liposomal AmB
LOS	late-onset sepsis
MIC	minimum inhibitory concentration
MRSA	methicillin-resistant staphylococcus aureus
ND	neurodevelopmental
NEC	necrotizing enterocolitis
NICU	neonatal intensive care unit
NMDA	n-methyl D-aspartate
PD	pharmacodynamics
PK	pharmacokinetics
PMA	postmenstrual age
PNA	postnatal age
RCT	randomized clinical trial
Spp.	species
VLBWIs	very low birth weight infants
WHO	World Health Organization
