# Supplementary material for: Antibiotics, Analgesic Sedatives, and Antiseizure Medications Frequently Used in Critically Ill Neonates: A Narrative Review
Source: Children (Basel). 2024 Jul 18;11(7):871. doi: 10.3390/children11070871 (PMC11275925; doi:10.3390/children11070871)
Supplement: Supplementary file 1 [file children-11-00871-s001.zip › children-3014065-supplementary.pdf]

Supplementary material

TABLE S1. Detailed presentation of selective antimicrobial medications commonly used in neonates.

Medication [references]	Mechanism of action / bactericidal spectrum	Main neonatal indications	Neonatal dosing regimen	Side effects
**AMPICILLIN**<br>(beta-lactam antibiotic classified as aminopenicillins)<br>[1–3]	- It inhibits bacterial cell wall synthesis by binding to the membrane-associated penicillin-binding proteins (PBPs), inhibiting the synthesis of peptidoglycan eventually leading to lysis and cell death.<br>- Broad spectrum bactericidal activity against susceptible Gram-positive (including *Strept.* spp., *Enterococcus faecalis, Listeria monocytogenes*) and Gram-negative (*E. coli, Hemophilus influenzae, Neisseria meningitidis, Proteus mirabilis and Salmonellae*).	- Empiric treatment of suspected EOS (including meningitis) combined with an aminoglycoside<br>- Targeted treatment of infections (bacteriemia, pneumonia, urinary tract infections, meningitis, endocarditis, gastrointestinal infections) caused by susceptible pathogens.	<u>FDA label</u><br>- For meningitis and septicemia<br>GA ≤ 34 wks, PNA ≤ 7 days: 50 mg/kg/dose q12<br>GA ≤ 34 wks, PNA: 8-28 days: 75 mg/kg/dose q12<br>GA> 34 wks, PNA ≤ 28 days: 50 mg/kg/dose q8<br><br><u>AAP recommendation</u><br>**-** For septicemia: as above<br>- For meningitis<br>GA ≤ 34 wks, PNA ≤ 7 days: 100 mg/kg q8<br>GA ≤ 34 wks, PNA: 8-28 days: 75 mg/kg q6<br>GA > 34 wks, PNA ≤ 28 days: 100 mg/kg q8	- Allergic reactions: maculopapular or urticarial rash, fever (rare in neonates)<br>- Diarrhea<br>- Neurotoxicity including seizures (with high concentrations reported in adults)<br>- Prolonged bleeding time with repeated doses.
**GENTAMICIN**<br>[4–11]	- Bactericidal activity by binding irreversibly to 30S subunit of bacterial ribosomes, inhibiting protein synthesis and leading to cell death.<br>- Potent bactericidal activity against Enterobacteriaceae (*E. coli, Klebsiella spp., Enterobacter cloacae, E. aerogenes, Providencia spp., Proteus spp., Morganella spp., Serratia spp.*), good activity against *Staph. aureus* (methicillin-resistant and vancomycin-intermediate and -resistant isolates), *P.*	- Empiric treatment of suspected EOS combined with ampicillin.<br>- Treatment of infections caused by susceptible aerobic gram-negative bacilli (e.g. *Pseudomonas, Klebsiella, E. coli*) used in combination usually with a β-lactam antibiotic.	- Several dosing regimens have been proposed by most studies and neonatal drug formularies recommending dosages of 4–5 mg/kg/dose and prolonged dosing intervals (24–48 h) for term and preterm neonates with GA, PMA and PNA being the main determinant of the dosing interval.<br>- TDM is strongly suggested in: therapy duration > 7 days, therapeutic hypothermia and renal impairment. (Measure trough concentrations before every dose. Target trough concentration: < 2 mg/L).<br><br><u>Australasian Neonatal Medicines Formulary (2021)</u><br>GA < 30 wks: 5 mg/kg q48<br>GA = 30 - 34^{+6} : 5 mg/kg q36	- Nephrotoxicity (renal tubular dysfunction with increased urinary losses of sodium, calcium, magnesium)<br>- Ototoxicity<br>- Hypersensitivity (very rare: rash, fever, eosinophilia, laryngeal oedema)<br>- Neuromuscular blockade (rare, only reported in adults; increased risk when used with neuromuscular blocking agents, opioid analgesics and massive transfusions with citrate anticoagulated blood and in patients with hypermagnesemia).

	aeruginosa and to a lesser extent *Acinetobacter baumannii*.		GA ≥ 35^{+0} : 5 mg/kg q24<br>- Treatment individualization can be achieved by measuring Gentamicin concentration at 22 h after the administration of the 2nd dose and subsequent dose interval is regulated according to drug levels as indicated:<br>≤ 1.2 mg/L: every 24 h after previous dose<br>1.3-2.6 mg/L: every 36 h<br>2.7-3.5 mg/L: every 48 h<br>> 3.6 mg/L: hold dose, repeat drug level 24h later<br>- Extension of dose interval by 12h in case of ibuprofen/indomethacin co-administration.	
**MEROPENEM** [4,12–14]	- It binds to PBPs, disrupting bacterial cell wall synthesis, leading to lysis and cell death.<br>- Broad spectrum bactericidal activity against i) Gram-negative pathogens: *Enterobacteriaceae*, ESBL- and AmpC-producing *Enterobacteriaceae*, *Haemophilus influenzae and Neisseria meningitidis, Pseudomonas aeruginosa, Acinetobacter baumannii, Burkholderia cepacian*.<br>ii) Gram-positive pathogens: *Staph. aureus* (methicillin/oxacillin-susceptible), *Staph. epidermidis* (oxacillin-susceptible), *Strept. pneumoniae* (including penicillin resistant strains) and *viridans group strept*.<br>iii) anaerobes (*Cl. difficile, Cl. perfringens*).	- Severe neonatal infections (e.g. septicaemia, complicated intra-abdominal, urinary tract, skin and skin structure infections, pneumonia, bacterial meningitis) due to multi drug resistant Gram-negative organisms.	**FDA LABEL**<br>**Dosage regimen for:**<br>A) intra-abdominal and non-CNS infections<br>GA < 32 wks, PNA < 2 wks: 20 mg/kg/dose q12<br>GA < 32 wks, PNA ≥ 2 wks: 20 mg/kg/dose q8<br>GA ≥ 32 wks, PNA < 2 wks: 20 mg/kg/dose q8<br>GA ≥ 32 wks, PNA ≥ 2 wks: 30 mg/kg/dose q8<br><br>B) CNS infections (off-label)<br>Data regarding appropriate dosing for neonatal CNS infections are lacking; suggested dose: 40 mg/kg/dose at the recommended age-specific dosing interval.	- Diarrhea, rash, vomiting, glossitis<br>- Hematologic abnormalities: agranulocytosis, neutropenia, leukopenia<br>- Elevated creatinine<br>- Elevated direct bilirubin, aspartate transaminase (AST), alanine aminotransferase (ALT).
**VANCOMYCIN** [4,15–23]	- Bactericidal agent which interferes with cell wall synthesis, inhibits RNA synthesis and alters plasma membrane function leading to cell death.<br><br>- Bactericidal spectrum: *Staphylococci* (including MRSA), *Streptococci*, *Enterococci*, *Diphtheroids*, *Listeria*	- Infections due to susceptible strains of gram-positive microbes; *Staphylococci* (including MRSA), *Streptococci, Enterococci, Diphtheroids, Listeria*	No consensus on optimal dosing and monitoring in neonates. Various dosing regimens have been recommended and/or used in relevant studies which are mainly based on neonatal age (GA, PMA, PNA), body weight and serum creatinine.<br><br>Most recently suggested dosing regimen by Australasian Neonatal Medicines Formulary (2021)	- □ephrotoxicity; ototoxicity; Rash and hypotension (red man syndrome): may appear rapidly and resolves within minutes to hours, by increasing the infusion time we eliminate the risk for subsequent doses; neutropenia (reported after administration for >3 wks).

<table>
<tr><td></td><td>monocytogenes, Actinomyces Bacillus spp.</td><td>monocytogenes, Actinomyces spp.</td><td>Standard dose: 15 mg/kg/dose (IV)
Consideration for giving a loading dose 20 mg/kg/dose in cases of severe sepsis, MRSA, bone infection, meningitis, endocarditis, although evidence is limited
<table>
<tr><th>PMA (wks)</th><th>PNA (days)</th><th>INTERVAL</th></tr>
<tr><td>< 30</td><td>0-2</td><td>q18 h</td></tr>
<tr><td></td><td>3+</td><td>q12 h</td></tr>
<tr><td>30^{+0}-36^{+6}</td><td>0-14</td><td>q12 h</td></tr>
<tr><td></td><td>15+</td><td>q8 h</td></tr>
<tr><td>37^{+0} -44^{+6}</td><td>0-7</td><td>q12 h</td></tr>
<tr><td></td><td>8+</td><td>q8 h</td></tr>
<tr><td>$\geq 45^{+0}$</td><td>0+</td><td>q6 h</td></tr>
</table></td><td>- TDM is strongly suggested: Target C_{trough} = 10-15 mg/kg. Measure C_{trough} immediately prior to 3rd dose with the exception of:
1. $<29^{+0}$ PMA wks – before 2nd dose,
2. therapeutic hypothermia – before 2nd dose
3. renal impairment – before 2nd dose
- Check concentration prior to the 4th dose after any change in dose or frequency. Once C_{trough} target is reached, measure C_{trough} every 3 days prior to consecutive doses.
- More frequent monitoring may be required in renal impairment, infants receiving other nephrotoxic drugs or suspected severe sepsis.</td></tr>
</table>

C_{trough} = Trough concentration; Cl, Clostridium; E. coli; ESBL, extensive-spectrum beta-lactamase; Escherichia coli; EOS, early-onset sepsis; GA, gestational age; h, hour(s); MRSA, Methicillin-resistant Staphylococcus aureus; PBPs, penicillin-binding proteins; PNA, postnatal age; PMA, postmenstrual age (PMA= GA+P□A); spp, species; TDM, therapeutic drug monitoring; wks, weeks.

TABLE S2. Detailed presentation of selective antifungal medications commonly used in neonates.

Medication [references]	Mechanisms of action / fungicide spectrum	Main neonatal indications	Neonatal dosing regimen	Side effects
**Amphotericin B Deoxycholate** (AmB-D) (Polyene) [24–33]	- Loss of cell membrane integrity by binding to ergosterol. The polyene-ergosterol complex creates pores in the fungal cell membrane, leading to electrolyte leakage, cell lysis and cell death. Potent and broad fungicidal activity.	- Treatment of invasive fungal infections by susceptible fungi including *Candida* spp., *Aspergillus* spp. and *Cryptococcus* spp.<br>- First-line therapy for neonatal IC including CNS infections.<br>- An alternative therapy of invasive aspergillosis in neonates.	1 mg/kg, IV, daily.	Nephrotoxicity (acute kidney injury and electrolyte-wasting tubular acidosis); Increased risk of nephrotoxicity in co-administration with other nephrotoxic drugs (vancomycin, aminoglycocides). Electrolyte disturbances: hypokalemia, hypomagnesaemia, hypocalcaemia. Hematological: anaemia, leukopenia, thrombocytopenia. Gastrointestinal: elevated liver enzymes, diarrhoea, vomiting. Thrombophlebitis at the injection site. Infusion-related reactions: fever, hypotension (rare in neonates). Skin rashes. Monitoring of renal function, liver function, electrolytes and full blood count.
**Liposomal Amphotericin B** (Polyenes) [24,26,27,33]	Same as AmB-D	- Same as AmB-D.<br>- An alternative therapy for neonatal IC with caution in the presence of urinary tract infections because of reduced renal excretion.<br>- Drug of choice for neonatal invasive aspergillosis.	3-5 mg/kg, IV, daily.	- Similar adverse events with AmB-D.<br>- Reduced toxicity as compared with AmB-D.<br>- Monitoring of renal function, liver function, electrolytes and full blood count.
**Fluconazole** (Triazole) [25,34–42]	- Inhibition of fungal cytochrome P450 activity and ergosterol synthesis, with accumulation of toxic sterols in the cell membrane leading to fungal cell membrane disruption, cell content leakage, lysis and cell death.	- Treatment of invasive infections by susceptible *Candida* species (minimal activity against *C. glabrata,* no activity against *C. kruseii),* mucosal candidiasis (oropharyngeal, oesophageal), cryptococcal meningitis.<br>- An alternative therapy of IC in neonates not been on fluconazole prophylaxis.<br>- A step-down treatment of Candida meningitis after response to initial therapy.<br>- Prophylaxis of Candida infections, in nurseries with high rates (>10%) of IC.	- Treatment:<br>Loading dose: 25 mg/kg, IV; maintenance dose: 12 mg/kg/d once a day, starting 24 hours after loading dose.<br>- Prophylaxis:<br>3-6 mg/kg every 72h for 4-6 weeks.	- Most common adverse effects: gastrointestinal irritation and elevation in liver function tests.<br>- Rare: Rash, leukopenia, neutropenia, agranulocytosis and thrombocytopenia. Weekly monitoring of liver enzymes.
**Micafungin** (Echinocandin)	- Inhibition of β(1-3)-glucan synthase activity preventing	- As salvage therapy of invasive Candida infections or in situations where resistance	- 4 to 10 mg/kg/day<br>- Clinical and non-	- Most common adverse events: infusion reactions and transient elevation of hepatic enzymes.

[43–47]	synthesis of the fungal cell wall. It exerts fungicidal activity against *Candida* spp. (even against *C. glabrata and C. kruseii* resistant to fluconazole).	or toxicity preclude the use of AmB-D or fluconazole.<br>- There are concerns regarding the penetration of echinocandins into the CSF. Insufficient data in neonates do not yet allow the recommendation of their use in CNS infections.	clinical studies indicate that higher dose of at least 10 mg/kg daily is likely needed for candidemia with meningoencephalitis.	- Hyponatremia, hypochloremia, hypokalemia, elevated creatinine, acute intravascular hemolysis, hemolytic anemia and hemoglobinuria, monocytosis, thrombocytopenia, fever, rash, diarrhoea, vomiting have been reported.

AmB, Amphotericin B; AmB-Deoxycholate, AmB-D; C, Candida; h, hour(s); C□S, central nervous system; h, hour(s); IC, invasive candidiasis; IV, intravenously; spp, species

References

1. Mukhopadhyay, S.; Wade, K.C.; Puopolo, K.M. Drugs for the Prevention and Treatment of Sepsis in the □ewborn. *Clin. Perinatol.* **2019**, *46*, 327–347, doi:10.1016/j.clp.2019.02.012.

2. Research, C. for D.E. and NIH Funded Pediatric Labeling Changes for Drugs Studied under the 409i Process. *FDA* **2024**. https://www.fda.gov/drugs/development-resources/nih-funded-pediatric-labeling-changes-drugs-studied-under-409i-process. (Accessed on 17 July 2024)

3. “American Academy of Pediatrics. In: Kimberlin DW, Barnett ED, Lynfield R, Sawyer MH, eds. Red Book: 2021 Report of the Committee on Infectious Diseases. Itasca, IL: American Academy of Pediatrics: 2021. Available online at: http://www.reddepadressolidarios.com/img/1rps_1634118322_a.pdf (accessed on 17 June 2024).

4. ANMF - Australasian Neonatal Medicines Formulary Available online: https://www.anmfonline.org/ (accessed on 22 April 2024).
5. Krause, K.M.; Serio, A.W.; Kane, T.R.; Connolly, L.E. Aminoglycosides: An Overview. *Cold Spring Harb. Perspect. Med.* **2016**, *6*, a027029, doi:10.1101/cshperspect.a027029.
6. The WHO AWaRe (Access, Watch, Reserve) Antibiotic Book Available online: https://www.who.int/publications/i/item/9789240062382 (accessed on 22 June 2024).

7. Hodiamont, C.J.; van den Broek, A.K.; de Vroom, S.L.; Prins, J.M.; Mathôt, R.A.A.; van Hest, R.M. Clinical Pharmacokinetics of Gentamicin in Various Patient Populations and Consequences for Optimal Dosing for Gram-Negative Infections: An Updated Review. *Clin. Pharmacokinet.* **2022**, *61*, 1075–1094, doi:10.1007/s40262-022-01143-0.
8. Van Maarseveen, E.M.; Sprij, A.; Touw, D.J. Extended-Interval Dosing of Gentamicin Aiming for a Drug-Free Period in Neonates: A Prospective Cohort Study. *Ther. Drug Monit.* **2016**, *38*, 402–406, doi:10.1097/FTD.0000000000000283.
9. Low, Y.S.; Tan, S.L.; Wan, A.S. Extended-Interval Gentamicin Dosing in Achieving Therapeutic Concentrations in Malaysian Neonates. *J. Pediatr. Pharmacol. Ther. JPPT* **2015**, *20*, 119–127, doi:10.5863/1551-6776-20.2.119.
10. Hollander, E.M.; van Tuinen, E.L.; Schölvinck, E.H.; Bergman, K.A.; Bourgonje, A.R.; Gracchi, V.; Kneyber, M.C.J.; Touw, D.J.; Mian, P. Evaluation of Dosing Guidelines for Gentamicin in Neonates and Children. *Antibiot. Basel Switz.* **2023**, *12*, 810, doi:10.3390/antibiotics12050810.
11. [illegible]eeli, H.; Hanna, [illegible].; Abduljalil, K.; Cusumano, J.; Taft, D.R. Application of Physiologically Based Pharmacokinetic-Pharmacodynamic Modeling in Preterm Neonates to Guide Gentamicin Dosing Decisions and Predict Antibacterial Effect. *J. Clin. Pharmacol.* **2021**, *61*, 1356–1365, doi:10.1002/jcph.1890.
12. Baldwin, C.M.; Lyseng-Williamson, K.A.; Keam, S.J. Meropenem: A Review of Its Use in the Treatment of Serious Bacterial Infections. *Drugs* **2008**, *68*, 803–838, doi:10.2165/00003495-200868060-00006.
13. Germovsek, E.; Lutsar, I.; Kipper, K.; Karlsson, M.O.; Planche, T.; Chazallon, C.; Meyer, L.; Trafojer, U.M.T.; Metsvaht, T.; Fournier, I.; et al. Plasma and CSF Pharmacokinetics of Meropenem in Neonates and Young Infants: Results from the NeoMero Studies. *J. Antimicrob. Chemother.* **2018**, *73*, 1908–1916, doi:10.1093/jac/dky128.
14. Chiusaroli, L.; Liberati, C.; Caseti, M.; Rulli, L.; Barbieri, E.; Giaquinto, C.; Donà, D. Therapeutic Options and Outcomes for the Treatment of Neonates and Preterms with Gram-Negative Multidrug-Resistant Bacteria: A Systematic Review. *Antibiot. Basel Switz.* **2022**, *11*, 1088, doi:10.3390/antibiotics11081088.
15. Wilbaux, M.; Fuchs, A.; Samardzic, J.; Rodieux, F.; Csajka, C.; Allegaert, K.; van den Anker, J.[illegible].; Pfister, M. Pharmacometric Approaches to Personalize Use of Primarily Renally Eliminated Antibiotics in Preterm and Term Neonates. *J. Clin. Pharmacol.* **2016**, *56*, 909–935, doi:10.1002/jcph.705.
16. Boscarino, G.; Romano, R.; Iotti, C.; Tegoni, F.; Perrone, S.; Esposito, S. An Overview of Antibiotic Therapy for Early- and Late-Onset Neonatal Sepsis: Current Strategies and Future Prospects. *Antibiot. Basel Switz.* **2024**, *13*, 250, doi:10.3390/antibiotics13030250.
17. Rybak, M.J.; Le, J.; Lodise, T.P.; Levine, D.P.; Bradley, J.S.; Liu, C.; Mueller, B.A.; Pai, M.P.; Wong-Beringer, A.; Rotschafer, J.C.; et al. Therapeutic Monitoring of Vancomycin for Serious Methicillin-Resistant Staphylococcus Aureus Infections: A Revised Consensus Guideline and Review by the American Society of Health-System Pharmacists, the Infectious Diseases Society of America, the Pediatric Infectious Diseases Society, and the Society of Infectious Diseases Pharmacists. *Am. J. Health-Syst. Pharm. AJHP Off. J. Am. Soc. Health-Syst. Pharm.* **2020**, *77*, 835–864, doi:10.1093/ajhp/zxaa036.
18. Dao, K.; Guidi, M.; André, P.; Giannoni, E.; Basterrechea, S.; Zhao, W.; Fuchs, A.; Pfister, M.; Buclin, T.; Csajka, C. Optimisation of Vancomycin Exposure in Neonates Based on the Best Level of Evidence. *Pharmacol. Res.* **2020**, *154*, 104278, doi:10.1016/j.phrs.2019.104278.

19. Mejías-Trueba, M.; Alonso-Moreno, M.; Herrera-Hidalgo, L.; Gil-Navarro, M.V. Target Attainment and Clinical Efficacy for Vancomycin in Neonates: Systematic Review. *Antibiot. Basel Switz.* **2021**, *10*, 347, doi:10.3390/antibiotics10040347.
20. Chen, Y.; Wu, D.; Dong, M.; Zhu, Y.; Lu, J.; Li, X.; Chen, C.; Li, Z. Population Pharmacokinetics of Vancomycin and AUC-Guided Dosing in Chinese Neonates and Young Infants. *Eur. J. Clin. Pharmacol.* **2018**, *74*, 921–930, doi:10.1007/s00228-018-2454-0.
21. Tseng, S.-H.; Lim, C.P.; Chen, Q.; Tang, C.C.; Kong, S.T.; Ho, P.C.-L. Evaluating the Relationship between Vancomycin Trough Concentration and 24-Hour Area under the Concentration-Time Curve in Neonates. *Antimicrob. Agents Chemother.* **2018**, *62*, e01647-17, doi:10.1128/AAC.01647-17.
22. Hill, L.F.; Clements, M.□.; Turner, M.A.; Donà, D.; Lutsar, I.; Jacqz-Aigrain, E.; Heath, P.T.; Roilides, E.; Rawcliffe, L.; Alonso-Diaz, C.; et al. Optimised versus Standard Dosing of Vancomycin in Infants with Gram-Positive Sepsis (NeoVanc): A Multicentre, Randomised, Open-Label, Phase 2b, Non-Inferiority Trial. *Lancet Child Adolesc. Health* **2022**, *6*, 49–59, doi:10.1016/S2352-4642(21)00305-9.
23. Jarugula, P.; Akcan-Arikan, A.; Munoz-Rivas, F.; Moffett, B.S.; Ivaturi, V.; Rios, D. Optimizing Vancomycin Dosing and Monitoring in □eonates and Infants Using Population Pharmacokinetic Modeling. *Antimicrob. Agents Chemother.* **2022**, *66*, e0189921, doi:10.1128/aac.01899-21.
24. Roberts, J.K.; Stockmann, C.; Constance, J.E.; Stiers, J.; Spigarelli, M.G.; Ward, R.M.; Sherwin, C.M.T. Pharmacokinetics and Pharmacodynamics of Antibacterials, Antifungals, and Antivirals Used Most Frequently in Neonates and Infants. *Clin. Pharmacokinet.* **2014**, *53*, 581–610, doi:10.1007/s40262-014-0147-0.
25. Pappas, P.G.; Kauffman, C.A.; Andes, D.R.; Clancy, C.J.; Marr, K.A.; Ostrosky-Zeichner, L.; Reboli, A.C.; Schuster, M.G.; Vazquez, J.A.; Walsh, T.J.; et al. Clinical Practice Guideline for the Management of Candidiasis: 2016 Update by the Infectious Diseases Society of America. *Clin. Infect. Dis. Off. Publ. Infect. Dis. Soc. Am.* **2016**, *62*, e1-50, doi:10.1093/cid/civ933.
26. Downes, K.J.; Fisher, B.T.; Zane, □.R. Administration and Dosing of Systemic Antifungal Agents in Pediatric Patients. *Paediatr. Drugs* **2020**, *22*, 165–188, doi:10.1007/s40272-020-00379-2.
27. Shirzadi, M.R. Lipsosomal Amphotericin B: A Review of Its Properties, Function, and Use for Treatment of Cutaneous Leishmaniasis. *Res. Rep. Trop. Med.* **2019**, *10*, 11–18, doi:10.2147/RRTM.S200218.
28. Lepak, A.J.; Andes, D.R. Antifungal Pharmacokinetics and Pharmacodynamics. *Cold Spring Harb. Perspect. Med.* **2014**, *5*, a019653, doi:10.1101/cshperspect.a019653.
29. Chen, S.C.; Sorrell, T.C.; Chang, C.C.; Paige, E.K.; Bryant, P.A.; Slavin, M.A. Consensus Guidelines for the Treatment of Yeast Infections in the Haematology, Oncology and Intensive Care Setting, 2014. *Intern. Med. J.* **2014**, *44*, 1315–1332, doi:10.1111/imj.12597.
30. Jeon, G.W.; Koo, S.H.; Lee, J.H.; Hwang, J.H.; Kim, S.S.; Lee, E.K.; Chang, W.; Chang, Y.S.; Park, W.S. A Comparison of AmBisome to Amphotericin B for Treatment of Systemic Candidiasis in Very Low Birth Weight Infants. *Yonsei Med. J.* **2007**, *48*, 619–626, doi:10.3349/ymj.2007.48.4.619.
31. López Sastre, J.B.; Coto Cotallo, G.D.; Fernández Colomer, B.; Grupo de Hospitales Castrillo □eonatal Invasive Candidiasis: A Prospective Multicenter Study of 118 Cases. *Am. J. Perinatol.* **2003**, *20*, 153–163, doi:10.1055/s-2003-40008.
32. Andrew, E.C.; Curtis, □.; Coghlan, B.; Cranswick, □.; Gwee, A. Adverse Effects of Amphotericin B in Children; a Retrospective Comparison of Conventional and Liposomal Formulations. *Br. J. Clin. Pharmacol.* **2018**, *84*, 1006–1012, doi:10.1111/bcp.13521.

33. Ascher, S.B.; Smith, P.B.; Watt, K.; Benjamin, D.K.; Cohen-Wolkowiez, M.; Clark, R.H.; Benjamin, D.K.; Moran, C. Antifungal Therapy and Outcomes in Infants with Invasive Candida Infections. *Pediatr. Infect. Dis. J.* **2012**, *31*, 439–443, doi:10.1097/INF.0b013e3182467a72.
34. Stockmann, C.; Constance, J.E.; Roberts, J.K.; Olson, J.; Doby, E.H.; Ampofo, K.; Stiers, J.; Spigarelli, M.G.; Sherwin, C.M.T. Pharmacokinetics and Pharmacodynamics of Antifungals in Children and Their Clinical Implications. *Clin. Pharmacokinet.* **2014**, *53*, 429–454, doi:10.1007/s40262-014-0139-0.
35. Odds, F.C.; Brown, A.J.P.; Gow, □.A.R. Antifungal Agents: Mechanisms of Action. *Trends Microbiol.* **2003**, *11*, 272–279, doi:10.1016/s0966-842x(03)00117-3.
36. Carmo, A.; Rocha, M.; Pereirinha, P.; Tomé, R.; Costa, E. Antifungals: From Pharmacokinetics to Clinical Practice. *Antibiot. Basel Switz.* **2023**, *12*, 884, doi:10.3390/antibiotics12050884.
37. Brammer, K.W.; Coates, P.E. Pharmacokinetics of Fluconazole in Pediatric Patients. *Eur. J. Clin. Microbiol. Infect. Dis. Off. Publ. Eur. Soc. Clin. Microbiol.* **1994**, *13*, 325–329, doi:10.1007/BF01974613.
38. Sheehan, D.J.; Hitchcock, C.A.; Sibley, C.M. Current and Emerging Azole Antifungal Agents. *Clin. Microbiol. Rev.* **1999**, *12*, 40–79, doi:10.1128/CMR.12.1.40.
39. Wade, K.C.; Benjamin, D.K.; Kaufman, D.A.; Ward, R.M.; Smith, P.B.; Jayaraman, B.; Adamson, P.C.; Gastonguay, M.R.; Barrett, J.S. Fluconazole Dosing for the Prevention or Treatment of Invasive Candidiasis in Young Infants. *Pediatr. Infect. Dis. J.* **2009**, *28*, 717–723, doi:10.1097/INF.0b013e31819f1f50.
40. Piper, L.; Smith, P.B.; Hornik, C.P.; Cheifetz, I.M.; Barrett, J.S.; Moorthy, G.; Hope, W.W.; Wade, K.C.; Cohen-Wolkowiez, M.; Benjamin, D.K. Fluconazole Loading Dose Pharmacokinetics and Safety in Infants. *Pediatr. Infect. Dis. J.* **2011**, *30*, 375–378, doi:10.1097/INF.0b013e318202cbb3.
41. Cook, A.; Ferreras-Antolin, L.; Adhisivam, B.; Ballot, D.; Berkley, J.A.; Bernaschi, P.; Carvalheiro, C.G.; Chaikittisuk, □.; Chen, Y.; Chibabhai, V.; et al. Neonatal Invasive Candidiasis in Low- and Middle-Income Countries: Data from the NeoOBS Study. *Med. Mycol.* **2023**, *61*, myad010, doi:10.1093/mmy/myad010.
42. Hornik, C.D.; Bondi, D.S.; Greene, □.M.; Cober, M.P.; John, B. Review of Fluconazole Treatment and Prophylaxis for Invasive Candidiasis in Neonates. *J. Pediatr. Pharmacol. Ther. JPPT Off. J. PPAG* **2021**, *26*, 115–122, doi:10.5863/1551-6776-26.2.115.
43. Menezes, R. de P.; Ferreira, I.C. da S.; Lopes, M.S.M.; de Jesus, T.A.; de Araújo, L.B.; Santos Pedroso, R.D.; Röder, D.V.D. de B. Epidemiological Indicators and Predictors of Lethality Associated with Fungal Infections in a NICU: A Historical Series. *J. Pediatr. (Rio J.)* **2024**, *100*, 267–276, doi:10.1016/j.jped.2023.11.005.
44. Taormina, G.; Gopinath, R.; Moore, J.; Yasinskaya, Y.; Colangelo, P.; Reynolds, K.; □ambiar, S. A Regulatory Review Approach for Evaluation of Micafungin for Treatment of Neonatal Candidiasis. *Clin. Infect. Dis. Off. Publ. Infect. Dis. Soc. Am.* **2021**, *73*, 2335–2340, doi:10.1093/cid/ciab025.
45. Heresi, G.P.; Gerstmann, D.R.; Reed, M.D.; van den Anker, J.□.; Blumer, J.L.; Kovanda, L.; Keirns, J.J.; Buell, D.□.; Kearns, G.L. The Pharmacokinetics and Safety of Micafungin, a Novel Echinocandin, in Premature Infants. *Pediatr. Infect. Dis. J.* **2006**, *25*, 1110–1115, doi:10.1097/01.inf.0000245103.07614.e1.

46. Smith, P.B.; Walsh, T.J.; Hope, W.; Arrieta, A.; Takada, A.; Kovanda, L.L.; Kearns, G.L.; Kaufman, D.; Sawamoto, T.; Buell, D.[illegible].; et al. Pharmacokinetics of an Elevated Dosage of Micafungin in Premature Neonates. *Pediatr. Infect. Dis. J.* **2009**, *28*, 412–415, doi:10.1097/INF.0b013e3181910e2d.
47. Benjamin, D.K.; Smith, P.B.; Arrieta, A.; Castro, L.; Sánchez, P.J.; Kaufman, D.; Arnold, L.J.; Kovanda, L.L.; Sawamoto, T.; Buell, D.[illegible].; et al. Safety and Pharmacokinetics of Repeat-Dose Micafungin in Young Infants. *Clin. Pharmacol. Ther.* **2010**, *87*, 93–99, doi:10.1038/clpt.2009.200.
